# *De novo* Transcriptome Analysis of Chinese Citrus Fly, *Bactrocera minax* (Diptera: Tephritidae), by High-Throughput Illumina Sequencing

**DOI:** 10.1371/journal.pone.0157656

**Published:** 2016-06-22

**Authors:** Jia Wang, Ke-Cai Xiong, Ying-Hong Liu

**Affiliations:** Institute of Entomology, College of Plant Protection, Southwest University, Chongqing, P. R. China; University of Georgia, UNITED STATES

## Abstract

The Chinese citrus fly, *Bactrocera minax* (Enderlein), is one of the most devastating pests of citrus in the temperate areas of Asia. So far, studies involving molecular biology and physiology of *B*. *minax* are still scarce, partly because of the lack of genomic information and inability to rear this insect in laboratory. In this study, *de novo* assembly of a transcriptome was performed using Illumina sequencing technology. A total of 20,928,907 clean reads were obtained and assembled into 33,324 unigenes, with an average length of 908.44 bp. Unigenes were annotated by alignment against NCBI non-redundant protein (Nr), Swiss-Prot, Clusters of Orthologous Groups (COG), Gene Ontology (GO), and Kyoto Encyclopedia of Genes and Genomes Pathway (KEGG) database. Genes potentially involved in stress tolerance, including 20 heat shock protein (Hsps) genes, 26 glutathione S-transferases (GSTs) genes, and 2 ferritin subunit genes, were identified. These genes may play roles in stress tolerance in *B*. *minax* diapause stage. It has previously been found that 20E application on *B*. *minax* pupae could avert diapause, but the underlying mechanisms remain unknown. Thus, genes encoding enzymes in 20E biosynthesis pathway, including Neverland, Spook, Phantom, Disembodied, Shadow, Shade, and Cyp18a1, and genes encoding 20E receptor proteins, ecdysone receptor (EcR) and ultraspiracle (USP), were identified. The expression patterns of 20E-related genes among developmental stages and between 20E-treated and untreated pupae demonstrated their roles in diapause program. In addition, 1,909 simple sequence repeats (SSRs) were detected, which will contribute to molecular marker development. The findings in this study greatly improve our genetic understanding of *B*. *minax*, and lay the foundation for future studies on this species.

## Introduction

Recently, the high-throughput sequencing technologies, referred to as the next-generation sequencing, such as Solexa/Illumina, SOLID/ABI, 454/Roche platform, has widely been used to generate large amounts of sequence data for characterizing genomes and transcriptomes, which have facilitated the studies on biological processes in organisms [1–3]. The transcriptome sequencing could serve as an efficient approach to obtain the genetic information of non-model species that lack genome database. Therefore, the transcriptomes of several insect species, such as *Bemisia tabaci* [4], *Liposcelis entomophila* [5], *Bactrocera dorsalis* [6], *Monochamus alternatus* [7], *Blattella germanica* [8], and *Chrysomya megacephala* [9], have been sequenced using next-generation sequencing to identify interesting genes and reveal gene expression patterns.

Another major benefit brought about by next-generation sequencing technology is the discovery of microsatellite markers simple sequence repeats (SSRs). Given the properties of high polymorphism and ease of scoring [10], the molecular marker SSRs have widely been used in population genetic and conservation studies, such as population size, kinship, bottlenecks, and migration rate [11]. The large-scale screen of SSRs necessitates the availability of abundant genetic information. The transcriptome sequencing meets this requirement and thus greatly facilitates the discovery of SSRs.

The Chinese citrus fly, *Bactrocera minax* (Enderlein), has been recognized as one of the most devastating pests of citrus in the temperate areas of Asia, including Nepal, India, Bhutan, and China [12–14]. The oligophagous *B*. *minax* specifically damages cultivated and wild species of citrus [15], causing the fruits to ripen prematurely and drop to the soil [16–19]. Given the economic importance, *B*. *minax* has increasingly aroused concerns in citrus-growing regions in China, thus the pertinent studies have been carried out. However, the researches were mainly focused on ecology, biology, and management of this pest [12,19–26]. Recently, studies involving molecular biology and physiology have increasingly been conducted [27–32], but they are still scarce at present partly due to the inability to rear this insect in the laboratory and the lack of genetic information.

One of the limiting factors to rearing *B*. *minax* in the laboratory is the long-lasting pupal stage, in which the diapause occurs. It has previously been shown that application of ecdysone 20-hydroxyecdysone (20E) on *B*. *minax* pupae can significantly advance the adult emergence [28]. However, the underlying mechanisms remain unknown. In insects, the 20E is synthesized from cholesterol under the manipulation of a set of genes, including *Neverland*, *Spook*, *Phantom*, *Disembodied*, *Shadow*, *Shade*, and degraded to 20-hydroxyecdysonoic acid by catalysis of Cyp18a1 [33,34]. The dynamics of 20E is essential for proper development of insects. Generally, the ecdysone signal in insect is amplified by a cascade of primary and secondary response genes that are activated by rising ecdysone levels [35]. The ecdysone receptor (EcR) and ultraspiracle (USP) have been demonstrated to be ecdysone receptors in the form of heterodimeric complex and mediate the biological activity of ecdysone [36–38]. In *B*. *minax*, these genes may also regulate the roles of 20E in diapause termination.

To survive the unfriendly environment during diapause, insects have to be tolerant to various abiotic and biotic stresses. Heat shock proteins (Hsps) are a group of well described proteins that are commonly expressed in response to these stresses [39]. Glutathione S-transferases (GSTs) comprise a family of enzymes best known for their ability to catalyze the conjugation of the reduced form of glutathione to substrates for the purpose of detoxification [40]. Ferritins are iron-binding proteins which play key roles in iron transport and storage to prevent oxidative damage caused by iron [41]. It has been found that Hsps [42], GSTs [43], and ferritins [44] involved in diapause programs in insects, which may also be the case for *B*. *minax*.

In this study, the Illumina sequencing was conducted to generate transcriptome dataset of *B*. *minax*. All assembled unigenes were annotated by BLASTx against databases of NCBI non-redundant protein (Nr), Swiss-Prot, the Kyoto Encyclopedia of Genes and Genomes (KEGG) database, Cluster of Orthologous Groups (COG), and gene ontology (GO). Subsequently, the genes encoding Hsps, GSTs, Ferritins, enzymes in ecdysone biosynthesis pathway, and ecdysone receptors were identified. The expression patterns of ecdysone-related genes were investigated across the developmental stages, and were compared between 20E-treated and untreated pupae to reveal the roles of these genes in 20E signaling. In addition, a large-scale screen of SSRs in *B*. *minax* was conducted based on the transcriptome data. Undoubtedly, the transcriptome dataset will be an invaluable resource for future studies on *B*. *minax*.

## Materials and Methods

### Ethics Statement

The owner of the orchard in Wulong County, Chongqing Municipality, China, provided permissions to collect the samples for our scientific research.

### Insects

To obtain a comprehensive transcriptome dataset of *B*. *minax*, samples at various developmental stages were prepared, including eggs, early- and late-instar larvae, early-, middle-, and late-stage pupae, and female and male adults. All samples were collected from an orchard (Latitude: N29°344', Longitude: E107°546') in Wulong County, Chongqing Municipality, China, and were stored in liquid nitrogen for subsequent RNA extraction.

### RNA isolation, library construction and Illumina sequencing

Total RNA was extracted from each sample using TRIZOL Reagent (Life technologies, Carlsbad, CA, US) according to the manufacturer's instructions. RNA quantity was assessed with NanoVue spectrophotometer (GE Healthcare Bio-Science, Uppsala, Sweden). The purity and integrity of RNA was checked on 1% agarose gel electrophoresis. Equal amount of RNA isolated from all samples was pooled for constructing cDNA library.

Poly (A) mRNA was isolated from total RNA using oligo (dT) magnetic beads. Mixed with fragmentation buffer, the mRNA was fragmented to 200–700 bp. These short fragments were transcribed to the first-strand cDNAs with random hexamer primers, followed by the second-strand cDNAs synthesis using DNA polymerase I (New England BioLabs, Ipswich, MA) and RNase H (Invitrogen, Carlsbad, CA). These cDNA fragments were purified and resolved with EB buffer for end repair, single nucleotide A (adenine) addition, and ligation of adaptors. The suitable fragments judged by agarose gel electrophoresis was collected and used as templates for PCR amplification. The cDNA library was sequenced on Illumina HiSeq^™^ 2000 using paired-end technology in a single run.

### Transcriptome *de novo* assembly and bioinformatics analysis

The raw reads produced by sequencing instrument were filtered to remove adaptors sequences, low-quality sequences with unknown nucleotides N, and reads with more than 20% low quality bases (base quality < 10) using the NGS QC toolkit package (version 2.3) [45], and to remove rRNA sequence using SortMeRNA [46]. The clean reads data has been deposited in the NIH Short Read Archive (SRA) database (Accession No. SRR1272962). The *de novo* assembly of clean reads was conducted using the short reads assembling program Trinity [47]. Briefly, clean reads with overlapping sequence were combined to form contigs. The reads were then mapped back to the contigs. The contigs from the same transcript were detected with paired-end reads and assembled using paired-end joining and gap-filling method. The sequences that cannot be extended on either end were defined as unigenes.

All assembled unigenes were first aligned to NCBI Nr database and Swiss-Prot with a cut-off E-value of 10^−5^ using BLASTx (http://www.ncbi.nlm.nih.gov/). Subsequently, GO annotation was performed using Blast2GO program, a universal tool for annotation and analysis in functional genomics research [48]. Unigenes were also aligned to the COG database to predict and clarify the gene functions [49]. Lastly, unigenes were assigned to KEGG pathways using the online KEGG Automatic Annotation Server (KAAS)(http://www.genome.jp/kegg/kaas/). The sequence direction of the unigenes was determined by the best alignment results from databases in the priority order of Nr, Swiss-Prot, KEGG, and COG.

### Microsatellite markers detection

Microsatellite markers simple sequence repeats (SSRs) were detected in unigenes which are longer than 1kb, using MicroSAtellite (MISA) (http://pgrc.ipk-gatersleben.de/misa/) [50]. The parameters were adjusted for identification of perfect di-, tri-, tetra-, penta-, and hexa-nucleotide motifs with a minimum of 6, 5, 5, 5, and 5 repeats, respectively.

### Identification and analysis of interesting genes

Unigenes putatively encoding Hsps, GSTs, enzymes in 20E biosynthesis pathway, and 20E receptors were identified by alignment against databases with a cut-off E-value < 10^−5^. The full open reading frames of interesting genes were then determined using DNAMAN version 6 (http://www.lynnon.com) and were further verified by protein BLAST results. The deduced protein sequences of interesting genes were aligned with their counterparts of other insect species. Subsequently, the phylogenetic tree was constructed based on the amino acid sequence alignment using Neighbor-joining (NJ) method in software MEGA4 [51]. The reliability of the branching was tested by performing bootstrap analysis with 1,000 replications.

### Expression patterns of identified 20E-related genes

To compare the expressions of identified 20E-related genes among developmental stages, the second- and third-instar larvae, the pre-, early-, middle-, late-, and post-diapause pupae, and the adults were collected. In addition, 20E-treated and untreated pupae were obtained through the method described by Wang et al. [28] to investigate the effect of 20E application on expression patterns of identified 20E-related genes. Total RNA was extracted from each sample using TRIZOL Reagent and the cDNA was synthesized using PrimeScript^™^ RT Master Mix (Perfect Real Time) Kit (Takara, Shiga, Japan). Subsequently, real-time PCR was conducted using SYBR Premix Ex Taq^™^ II Kit (Takara) to calculate the relative expression of each gene. The specific primers for real-time PCR were shown in S1 Table. Three biological and technical replicates were performed for each treatment. Relative expression of each gene among developmental stages was compared by one-way analysis of variance (ANOVA) with Tukey’s post hoc test for difference among all pairs of test variants, while that between 20E-treated and untreated individuals was compared by two tailed, unpaired t-test. All data was analyzed using software SPSS version 22 (IBM Corp., Armonk, NY).

## Results and Discussion

### Illumina sequencing and *de novo* assembly

About 5.07 Gb raw data, including 25,088,946 raw reads, was generated from Illumina sequencing platform in a single run. After filtration, a total number of 20,928,907 clean reads, encompassing 4.227 Gb sequencing data, were assembled into 941,005 contigs with a mean length of 80.97 bp. The contigs were further assembled into 33,324 unigenes with a mean length of 908.44 bp (Table 1), which is much longer than that obtained from many other species, such as *L*. *entomophila* [5], *B*. *dorsalis* [6], and *M*. *alternatus* [7], indicating the efficient performance of sequencing and assembly in this study. The saturation curve illustrated that the sequencing data were saturated and sufficient for subsequent analysis (S1 Fig). This Transcriptome Shotgun Assembly project has been deposited at DDBJ/EMBL/GenBank under the accession GBEY00000000. The version described in this paper is the first version, GBEY01000000. Among all unigenes, 8,357 (25.08%) and 3,795 (11.39%) unigenes are longer than 1,000 and 2,000bp, respectively, and only 9960 (29.89%) unigenes are shorter than 300bp (S2 Fig), demonstrating the effectiveness of Illumina sequencing technology in rapidly capturing a large portion of the transcriptome and providing a sequence basis for future studies.

**Table 1:** Summary of Illumina transcriptome assembly for *Bactrocera minax*.

sequencing	
Raw data (G)	5.07
Total number of raw reads	25,088,946
Low quality data percentage (%)	13.54
rRNA percentage (%)	3.04
Clean data (G)	4.227
Total number of clean reads	20,928,907
Q20 percentage (%)	99.19
Q30 percentage (%)	95.32
GC percentage (%)	43.71
N percentage (%)	0.00
Number of contigs	941,005
Length of contigs (bp)	76,189,241
N50 length of contigs (bp)	102
Mean length of contigs (bp)	80.97
Number of unigenes	33,324
Length of unigenes (bp)	30,272,977
N50 length of unigenes (bp)	1733
Mean length of unigenes (bp)	908.44

### Sequence annotation

A total of 15,735 (47.22%) unigenes were successfully aligned to public protein databases with a cut-off E-value of 1.0E^-5^. As a close species to *B*. *minax*, the model species fruit fly *Drosophila melanogaster* has been subjected to transcriptomic analysis and 17,564 genes were annotated [52]. The similar number of annotation between these two studies implies that the high-quality *B*. *minax* transcriptome data was obtained. The remaining unigenes failed to acquire annotation, probably because they specifically express in *B*. *minax*, correspond to untranslated regions, or were assembled incorrectly.

**Fig 1:**
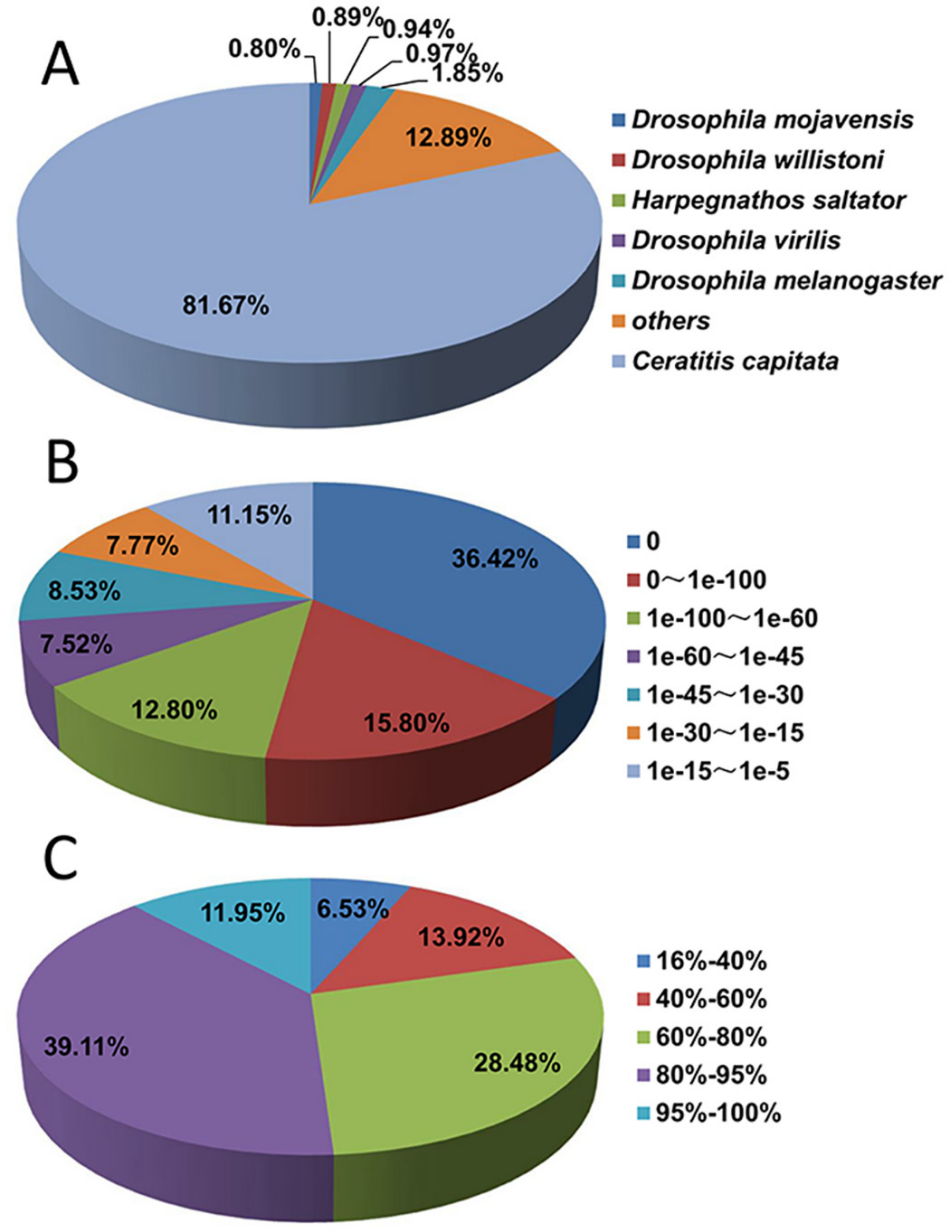
Homology search against Nr database for *Bactrocera minax* transcriptome unigenes. (A) Distribution of species of top BLAST hit. (B) Distribution of E-value of top BLAST hit with a cut-off E-value of 1.0E^-5^. (C) Distribution of similarity of top BLAST hit.

A total of 15,677 unigenes had hit against Nr database, the vast majority of these unigenes (81.67%) have top matches with genes from the Mediterranean fruit fly *ceratitis capitata*, and followed by fruit fly *Drosophila melanogaster* (1.85%), *Drosophila virilis* (0.97%) *Drosophila willistoni* (0.89%) and *Drosophila mojavensis* (0.80%) (Fig 1A). Among all annotated unigenes, 72.55% have significant homology with an E-value <10^−45^ (Fig 1B) and 51.06% have a similarity higher than 80% (Fig 1C).

**Fig 2:**
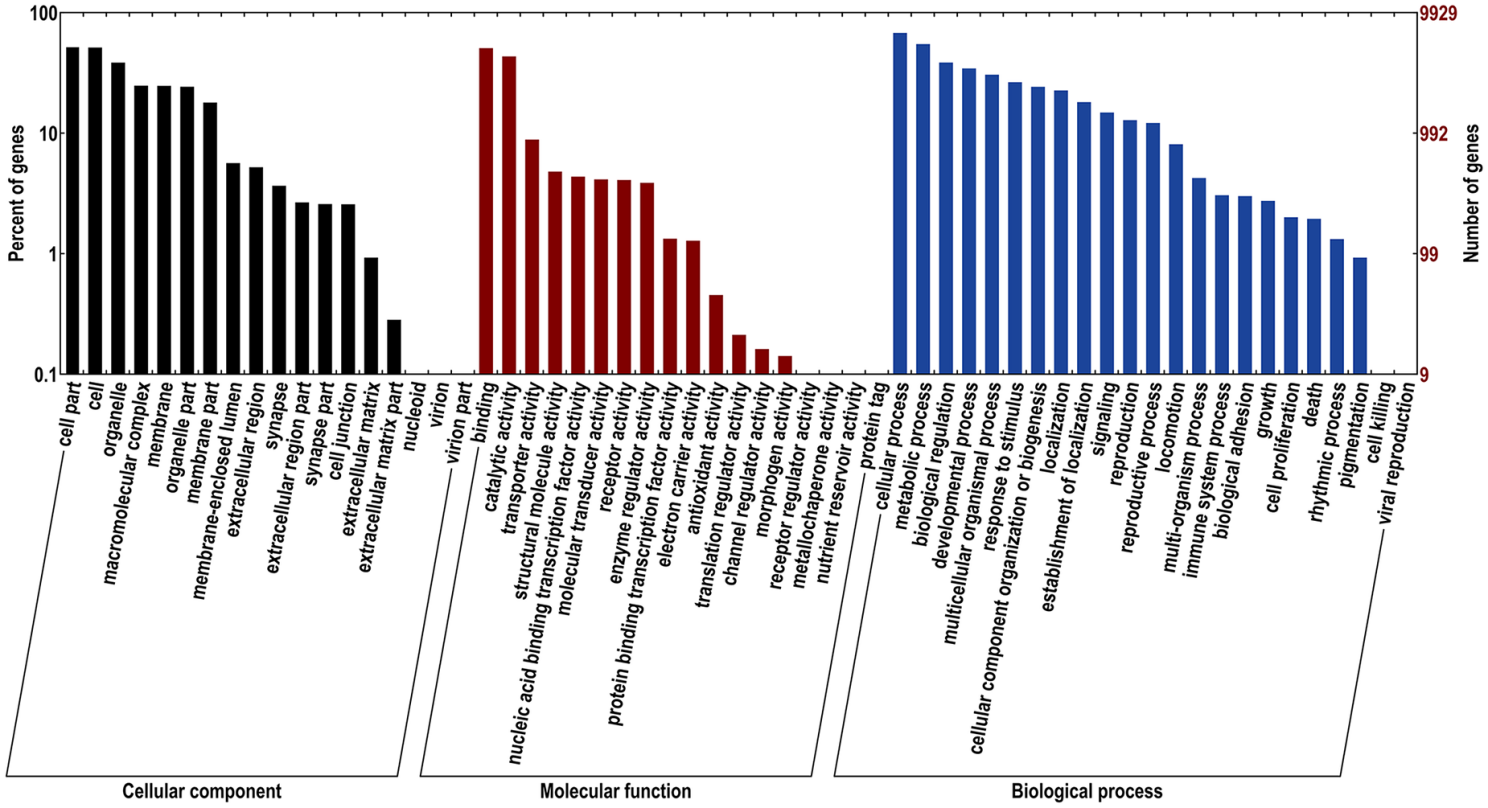
Gene Ontology (GO) classification of *Bactrocera minax* transcriptome unigenes.

GO is a standardized gene functional classification system that provides a structured and controlled vocabulary to predict gene functions [53]. Based on their similarity to genes with known functions, 9,950 unigenes were assigned at least one GO term, and 76,291 GO terms in total (Table 2). These GO terms fall into three main categories, ‘Cellular component’, ‘Molecular function’, and ‘Biological process’, which include 18, 18, and 23 subcategories, respectively (Fig 2). Many unigenes were assigned more than one GO terms, indicating they could be involved in various physiological and biochemical processes. Among three main categories, ‘Biological process’ accounts for the largest proportion of GO terms (38,159, 50.02%), followed by ‘Cellular component’ (25,451, 33.36%), and ‘Molecular function’ (12,681, 16.62%). In ‘Cellular component’ category, the ‘cell part’ (5,116, 6.71%) and ‘cell’ (5,089, 6.67%) are the most abundant. In ‘Molecular function’ category, the ‘binding’ (5,030, 6.59%) and ‘catalytic activity’ (4,295, 5.63%) are highly represented. In ‘Biological process’ category, the ‘cellular process’ (6,726, 8.82%) and ‘metabolic process’ (5,439, 7.13%) are assigned the most frequently (S2 Table). To program diapause in insect, several signaling pathways are activated. For example, the insulin signaling plays vital roles in cell cycle and developmental regulation, lifespan extension, suppressed metabolism and fat hypertrophy, and enhanced stress tolerance in insect diapause [54]. In the present study, 45 unigenes were assigned with 12 insulin-related GO terms (S3 Table). These genes may participate in the programming of diapause in *B*. *minax*. Moreover, the ‘response to stimulus’ is processes that causes changes in state and activity of cells or organisms as a result of stimulus, such as cold or hypoxia/anoxia stress. Genes assigned with this GO term may help diapausing *B*. *minax* pupae survive the harsh environment in winter.

**Table 2:** Summary of annotation for *Bactrocera minax* transcriptome unigenes.

Annotated database	No. of unigenes hit (%)	300–1000 bp	>1000 bp
Nr	15,677 (47.04)	6,037	7,629
Swiss-Prot	11,412 (34.25)	3,641	6,857
GO	9,950 (29.86)	3,233	5,705
COG	4,145 (12.44)	1,035	2,927
KEGG	4,295 (12.89)	1,261	2,726
All Annotated	15,735 (47.22)	6,058	7,632

**Fig 3:**
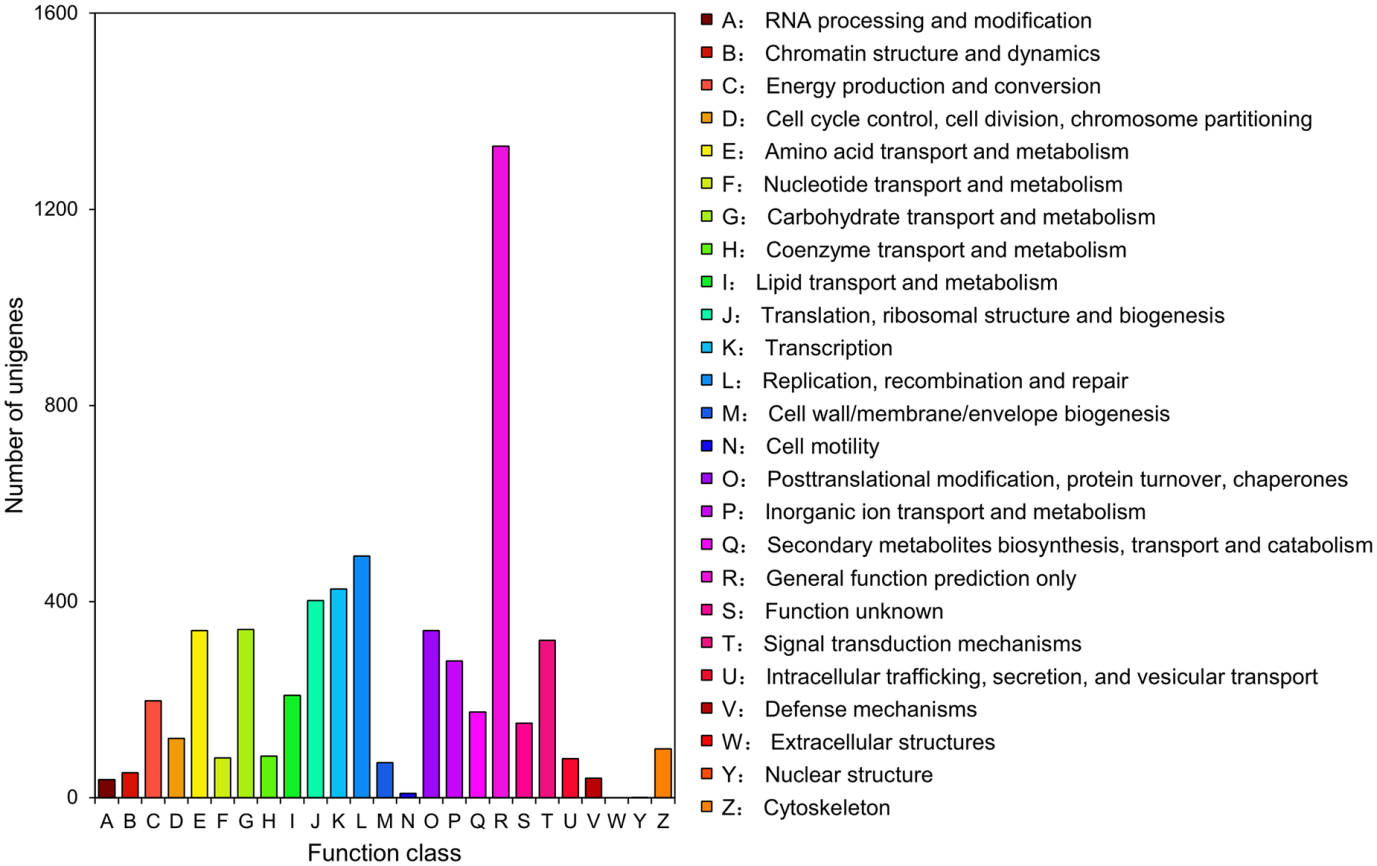
Clusters of Orthologous Group (COG) function classification of *Bactrocera minax* transcriptome unigenes.

In addition, all unigenes were subjected to alignment against the COG database for functional prediction and classification. In total, 4,145 unigenes could be assigned to COG classification and were classified into 24 COG categories (Table 2; Fig 3). Among all these categories, ‘General function prediction only’ (1,329, 32.06%) represented the largest group, followed by ‘Replication, recombination and repair’ (493, 11.89%), ‘transcription’ (426, 10.28%), and ‘Translation, ribosomal structure and biogenesis’ (402, 9.70%). Interestingly, no unigene was assigned to ‘Extracellular structures’, and only one unigene was assigned to ‘Nuclear structure’.

The KEGG pathway assignment was also performed for all assembled unigenes to categorize gene functions with the focus on biochemical pathways [55]. A total of 4,295 unigenes were annotated against KEGG database and were assigned to 271 pathways except those related to human diseases. The most enriched pathway is ‘Metabolic pathway’ (543, 12.64%), followed by ‘Biosynthesis of secondary metabolites’ (159, 3.70%), ‘Biosynthesis of antibiotics’ (120, 2.79%), and ‘RNA transport’ (110, 2.56%) (S3 Fig). It is well known that the endocrine hormones control the diapause program [56]. The prothoracicotropic hormone (PTTH) receptor signaling transduction [57] and ecdysone biosynthesis [56] are closely related to diapause termination, which involves several KEGG pathways, including ‘MAPK signaling pathway’ (Ko04010), ‘Wnt signaling pathway’ (Ko04310), ‘MAPK signaling pathway—fly’ (Ko04013), ‘mTOR signaling pathway’ (Ko04150), ‘Calcium signaling pathway’ (Ko04020), ‘Steroid biosynthesis’ (Ko00100), ‘Steroid hormone biosynthesis’ (Ko00140), ‘Terpenoid backbone biosynthesis’ (Ko00900), ‘Insect hormone biosynthesis’ (Ko00981), and ‘Insulin signaling pathway’ (Ko04910). Many unigenes belonging to these pathways were identified in *B*. *minax* transcriptome. The KEGG pathway assignment will be helpful for predicting the functions of *B*. *minax* genes, and will contribute to the further research on relevant metabolic pathways and biological processes.

### Simple sequence repeats discovery

Currently, only a few SSRs of *B*. *minax* were isolated in the previous study [58]. In this study, the SSRs were detected in unigenes which are longer than 1 Kb. A total of 1,909 SSRs were identified in *B*. *minax* transcriptome. Among all SSRs, trinucleotide repeats accounted for largest proportion (63.12%), followed by dinucleotide repeats (32.48%), trannucleotide repeats (3.46%), hexanucleotide repeats (0.63%), and pentanucleotide repeats (0.31%). Most of these SSRs have the number of repeats under 8 times (Table 3). Among all repeat types, AAC/GTT (29.18%), AC/GT (15.92%), AT/AT(12.94%), AGC/CTG (10.58%), and ACC/GGT (6.29%) are the most abundant ones (S4 Table). The SSRs identified in this study considerably enlarged the SSRs dataset of *B*. *minax* and they would be invaluable for the future studies related to population genetic structure, such as genetic variation and gene flow.

**Table 3:** Summary of simple sequence repeat (SSRs) types identified in *Bactrocera minax* transcriptome unigenes.

SSR motifs	No. of repeats									Total	Percentage (%)
	5	6	7	8	9	10	11	12	>12		
Dinucleotide	**-**	284	116	99	49	36	31	3	2	620	32.48
Trinucleotide	649	351	184	17	1	3	0	0	0	1,205	63.12
Trannucleotide	54	6	1	3	0	2	0	0	0	66	3.46
Pentanucleotide	4	2	0	0	0	0	0	0	0	6	0.31
Hexanucleotide	9	2	1	0	0	0	0	0	0	12	0.63
Total	716	645	302	119	50	41	31	3	2	1,909	

### Identification of heat shock protein genes

The heat shock proteins (Hsps) are known as stress proteins and molecular chaperones with functions of preventing irreversible denaturation of substrate proteins and promoting protein folding, degradation, disaggregation, and cell localization. They are important elements in stress response system at the cellular level when exposed to a wide variety of abiotic and biotic stressors, such as heat shock, cold, desiccation, starvation, anoxia, oxidation, osmotic stress, environmental contaminants, bacteria, and virus [39,42,59]. Moreover, Hsps have also been found employed during diapause, but the expression patterns of various Hsps in different species are inconsistent, even opposite [30,42,60].

**Fig 4:**
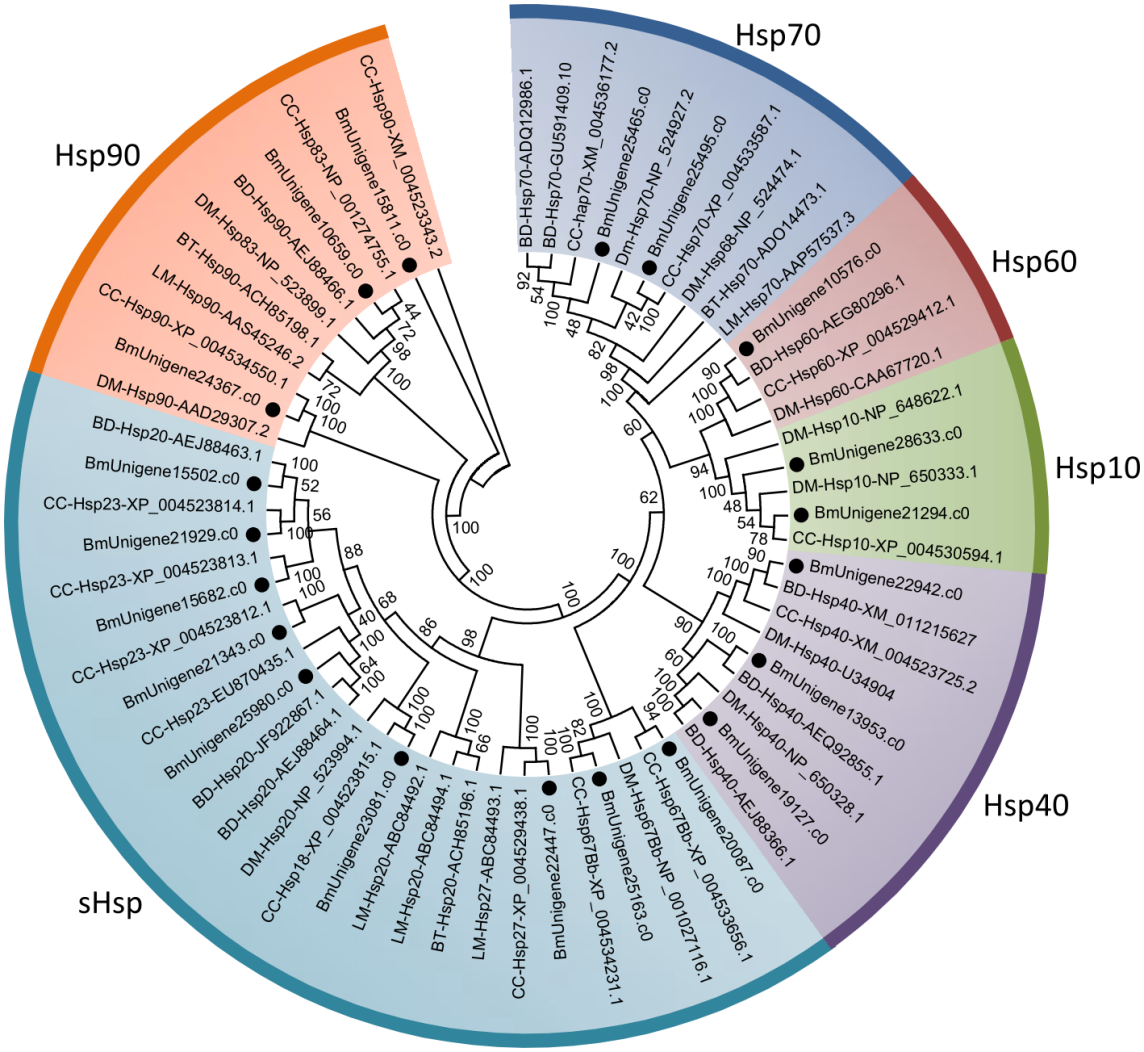
Neighbour-joining phylogenetic analysis of heat shock protein (Hsps) genes from *Bactrocera minax* (●) and other insects. BD, *Bactrocera dorsalis*. BT, *Bemisia tabaci*. CC, *Ceratitis capitata*. DM, *Drosophila melanogaster*. LM, *Locusta migratoria*. Numbers at each branch node represent the values given by bootstrap analysis.

Hsps represent a super gene family and can be divided into several families based on the molecular weight and homology [39]. In this study, a total of 23 unigenes putatively encoding Hsps were identified by alignment against databases. After manually removing short sequences, 20 unigenes containing full open reading frame were selected for subsequent analysis (S5 Table). Phylogenetic analysis of these Hsps indicated that they were divided into 6 families, Hsp90, Hsp70, Hsp60, Hsp40, Hsp10, and small Hsps (sHsps), encompassing 3, 2, 1, 3, 2, and 9 unigenes, respectively (Fig 4). The features and functions of Hsps vary with families. Briefly, Hsp90s activate and stabilize a wide variety of cytosolic proteins, which involve in important cellular pathways, such as signal transduction, intracellular transport, and protein degradation [61]. Hsp70s are structurally and functionally conserved and respond to stress by tightly binding its protein substrates and preventing them from denaturation or aggregation [62]. Hsp60 family is a group of multi-functional proteins implicated in several cellular processes, including stress response, amino acid transport, signal transduction, replication and transmission of mitochondrial DNA, and cellular metabolism [63–66]. Hsp40s, also known as DnaJs, interact with Hsp70 in J domain and regulate the ATPase activity of Hsp70s in several cellular processes [67]. Hsp40s usually work in conjunction with Hsp60s [68]. sHsps, with molecular weight ranging from 12 to 43 kDa, are known to bind to the non-native substrate proteins and prevent them from forming irreversible aggregations under stress conditions [69]. The roles of identified Hsps in the stress tolerance of diapausing *B*. *minax* remains largely unknown and necessitates further investigation.

### Identification of glutathione S-transferase genes

**Fig 5:**
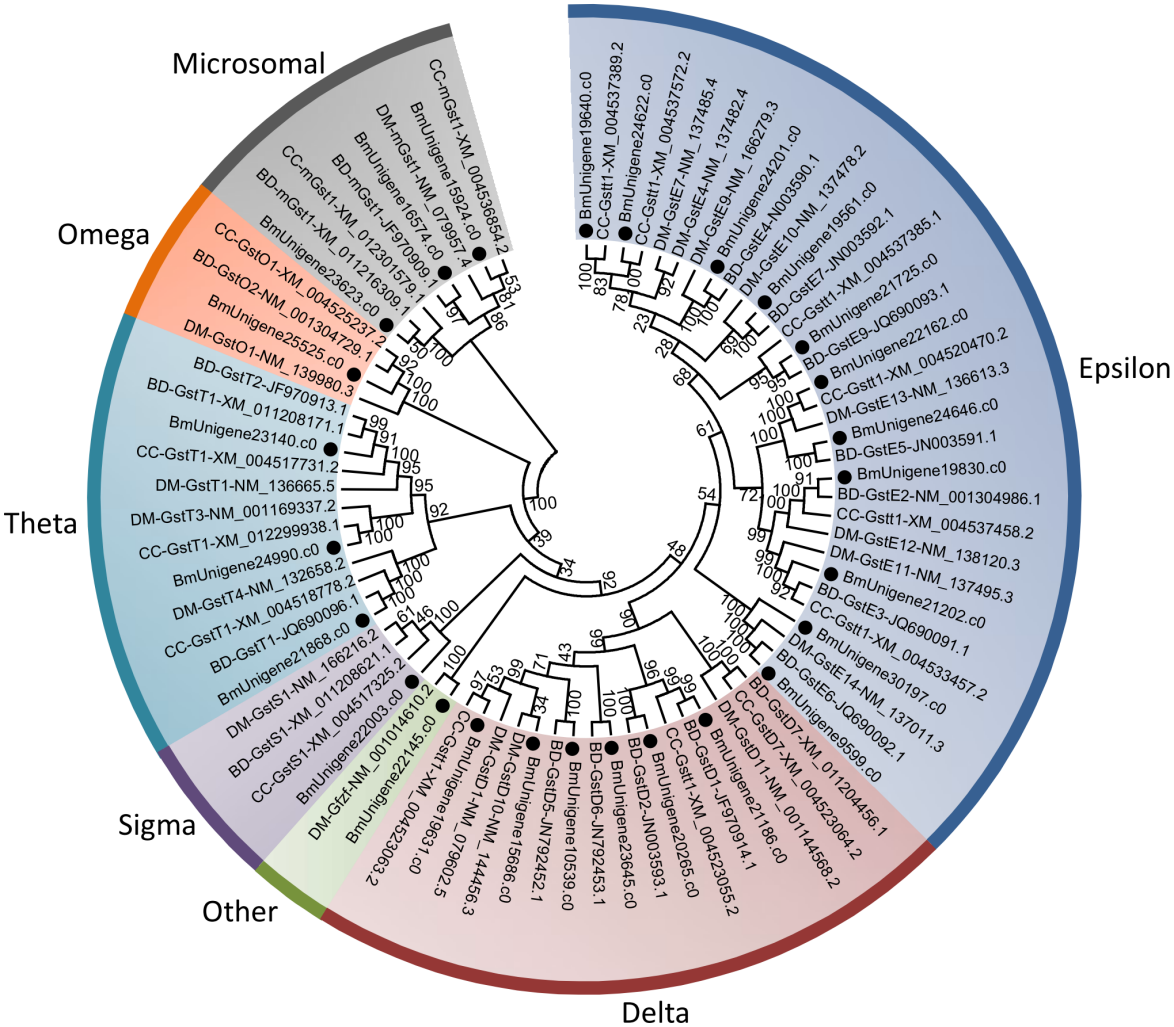
Neighbour-joining phylogenetic analysis of Glutathione S-transferase (GSTs) genes from *Bactrocera minax* (●) and other insects. BD, *Bactrocera dorsalis*. CC, *Ceratitis capitata*. DM, *Drosophila melanogaster*. Numbers at each branch node represent the values given by bootstrap analysis.

GSTs are a family of enzymes that involved in many cellular physiological activities, such as detoxification of endogenous and xenobiotic compounds, intracellular transport, biosynthesis of hormones and protection against oxidative stress [40,70]. A total of 27 unigenes encoding GSTs were identified in *B*. *minax* by alignment against databases, and 26 putative GSTs genes were manually selected for analysis after removing short unigenes (S6 Table). In insects, GSTs fall into several major subclasses: delta, epsilon, omega, sigma, theta, zeta, microsomal and others. Delta and epsilon are two unique classes to insects [70,71]. In this study, 26 unigenes were assigned to seven classes, including delta (6), epsilon (11), omega (1), sigma (1), theta (3), microsomal (3) and others (1). No GST belonging to zeta class was identified (Fig 5). Delta and epsilon occupy over 50% of the entire GSTs, which is consistent with previous studies from other dipteran insects [72]. It has been demonstrated that delta and epsilon classes correlate with detoxification and adaptation to environmental selection pressures [72,73]. The expansion of these classes may help *B*. *minax* survive the poisonous or harsh environments. Moreover, the GST levels have been found up-regulated in diapausing insects, which is speculated to protect individuals from oxidative damage as diapausing insects commonly experience hypoxia/anoxia stress [43,74,75]. The identification of genes encoding GSTs is conducive to understanding their potential roles in stress tolerance in *B*. *minax* diapause.

### Identification of ferritin genes

**Fig 6:**
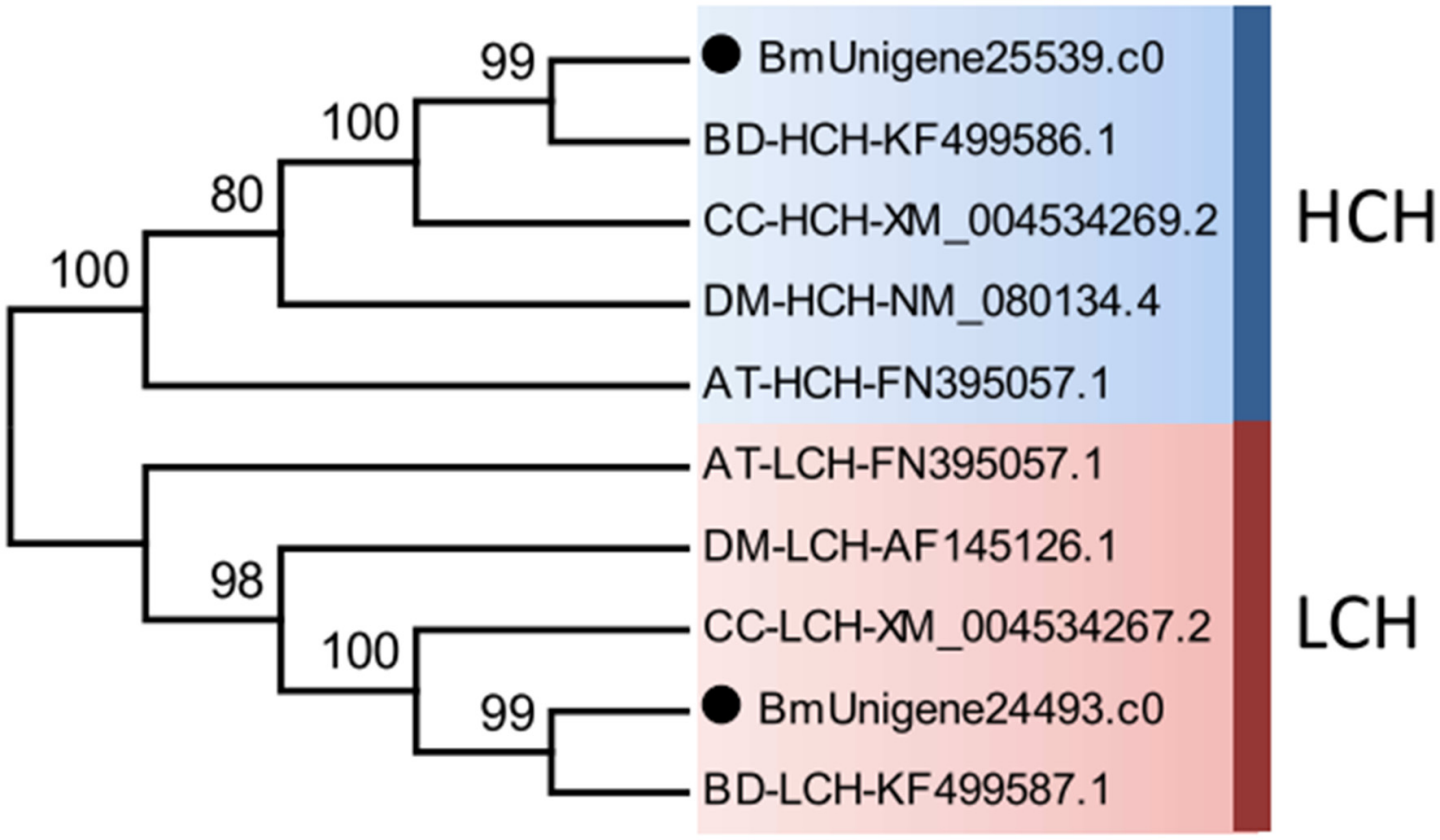
Neighbour-joining phylogenetic analysis of two ferritin subunit genes, heavy chain homologs (HCH) and light chain homologs (LCH), from Bactrocera minax (●) and other insects. AT, *Asobara tabida*. BD, *Bactrocera dorsalis*. CC, *Ceratitis capitata*. DM, *Drosophila melanogaster*. Numbers at each branch node represent the values given by bootstrap analysis.

Iron is not only an indispensable micronutrient, but also a potential toxin in organisms. On one hand, organisms must intake sufficient iron for various physiological processes [76]. On the other hand, organisms must avoid oxidative damage to biomolecules caused by the potential toxic properties of iron [77]. Ferritins are iron-binding proteins and play key roles in iron transport and storage to achieve iron homeostasis in organisms [41]. The active insect ferritin is a complex formed between two types of secretory subunits, a mammalian heavy chain homologue (HCH) that preserves the ferroxidase centers, and a mammalian light chain homologue (LCH) that contains the nucleation center for the formation of ferrihydrite iron core [78]. Insect HCH and LCH genes are arranged ‘head to head” and transcribed in opposite directions [79]. It has been found that ferritin genes expressed higher in diapause-destined insects, probably to resist oxidative stress caused by iron [44]. That may also be the case for *B*. *minax*. In this study, both HCH and LCH ferritin subunits of *B*. *minax* were identified by alignment against databases (S7 Table) and verified by phylogenetic analysis (Fig 6). Both HCH and LCH preserve signal peptide, C residues involved in inter- and intra-subunit disulfide bonds, and residues engaged in the salt bridges and pi-cation interactions. Only HCH, however, preserves all ferroxidase centers (S4 Fig).

### Identification of 20E-related genes

**Fig 7:**
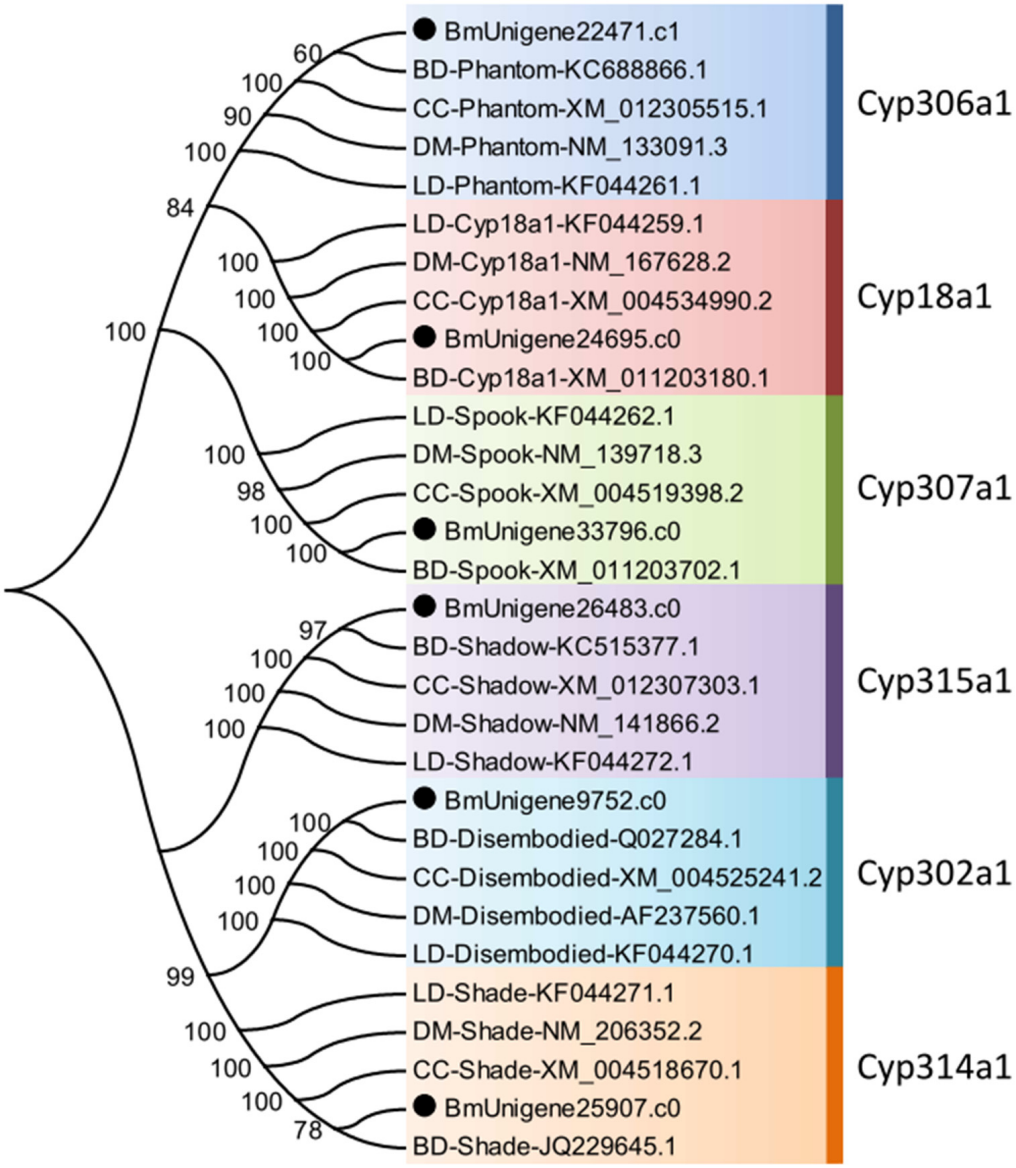
Neighbour-joining phylogenetic analysis of cytochrome P450 genes in ecdysone biosynthesis pathway from *Bactrocera minax* (●) and other insects. BD, *Bactrocera dorsalis*. CC, *Ceratitis capitata*. DM, *Drosophila melanogaster*. LD, *Leptinotarsa decemlineata*. Numbers at each branch node represent the values given by bootstrap analysis.

Ecdysone 20E is the key regulator of molting, reproduction, and diapause in insects. The biosynthesis of 20E is mediated by *Neverland*, Halloween genes, and *Cyp18a1*. Neverland, a conserved Rieske oxygenase, is responsible for the conversion of cholesterol to 7-dehydrocholesterol, which is the first critical catalytic step in the 20E biosynthesis pathway [80]. Halloween genes are a set of genes encoding cytochrome P450 enzymes, including Spook/Cyp307A1, Spookier/Cyp307a2, Phantom/Cyp306a1, Disembodied/Cyp302a1, Shadow/Cyp315a1, and Shade/Cyp314a1. Halloween genes are responsible for 20E biosynthesis from 7-dehydrocholesterol [33]. *Cyp18a1* encodes a cytochrome P450 enzyme with 26-hydroxylase activity, catalyzing the conversion of 20E to 20-hydroxyecdysonoic acid. The degradation of 20E is essential for proper development of insects [34]. Unigenes encoding *Neverland*, Halloween genes, and *Cyp18a1* in *B*. *minax* were identified by alignment against databases (S8 Table), and verified by phylogenetic analysis (Fig 7).

**Fig 8:**
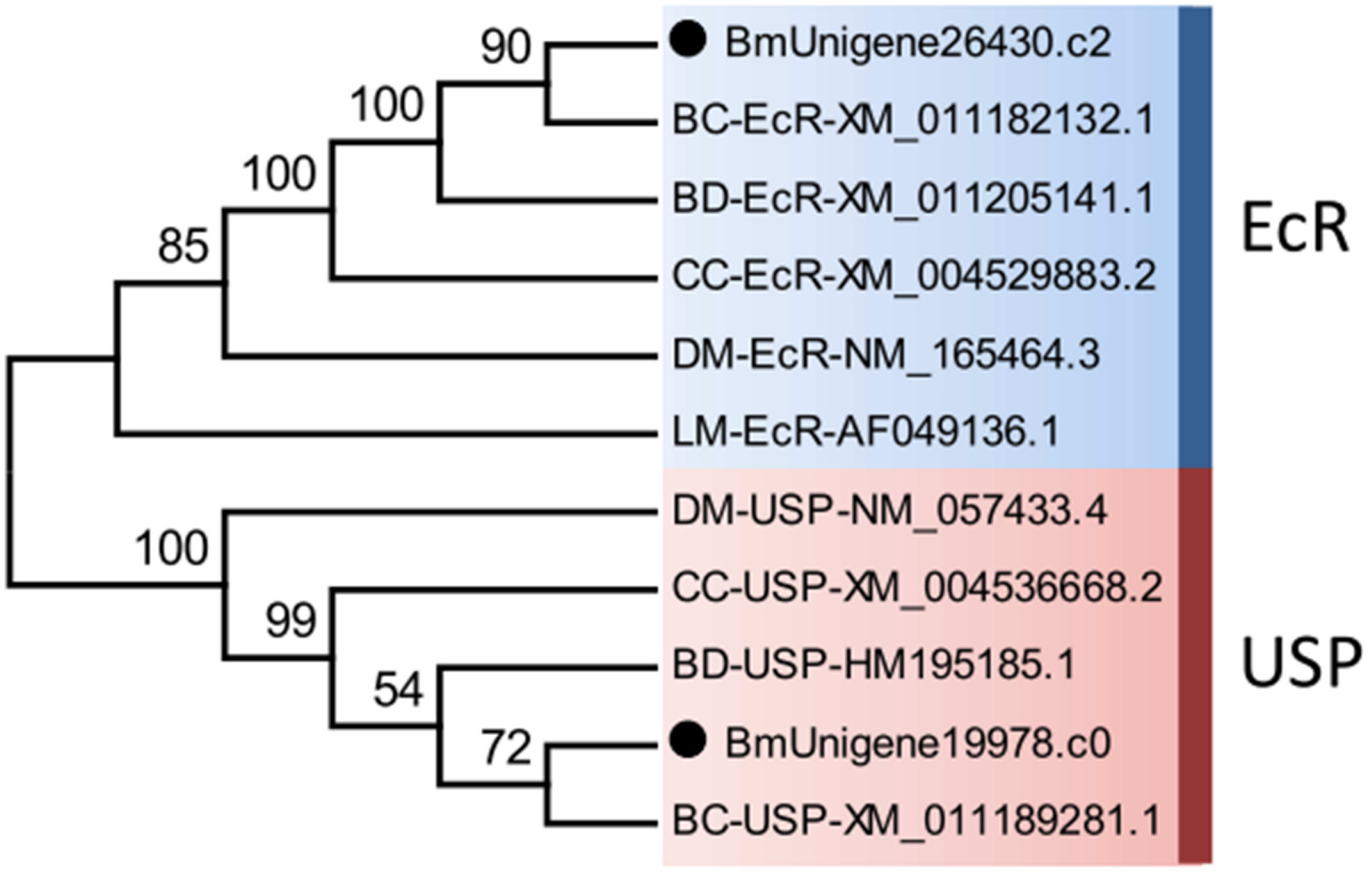
Neighbour-joining phylogenetic analysis of ecdysone receptor (EcR) and ultraspiracle (USP) genes from *Bactrocera minax* (●) and other insects. BC, *Bactrocera cucurbitae*. BD, *Bactrocera dorsalis*. CC, *Ceratitis capitata*. DM, *Drosophila melanogaster*. LM, *Locusta migratoria*. Numbers at each branch node represent the values given by bootstrap analysis.

The ecdysone receptor is a heterodimeric complex consisting of two proteins, EcR and USP, which are members of nuclear receptor superfamily, and the insect orthologs of the mammalian farnesoid X receptor (FXR) and retinoid X receptor (RXR) proteins, respectively [36–38]. Upon binding to the ecdysone receptor, the 20E/receptor complex binds to ecdysone-response elements in the promotor region of target genes and activates transcription [81]. Unigenes encoding EcR and USP in *B*. *minax* were identified by alignment against databases (S8 Table) and verified by phylogenetic analysis (Fig 8). We previously found that 20E application on *B*. *minax* pupae advanced adults emergence [28]. Therefore, the identification of these 20E-related genes would contribute to revealing the mechanisms underlying 20E induced diapause termination in *B*. *minax*.

### Expression patterns of identified 20E-related genes

**Fig 9:**
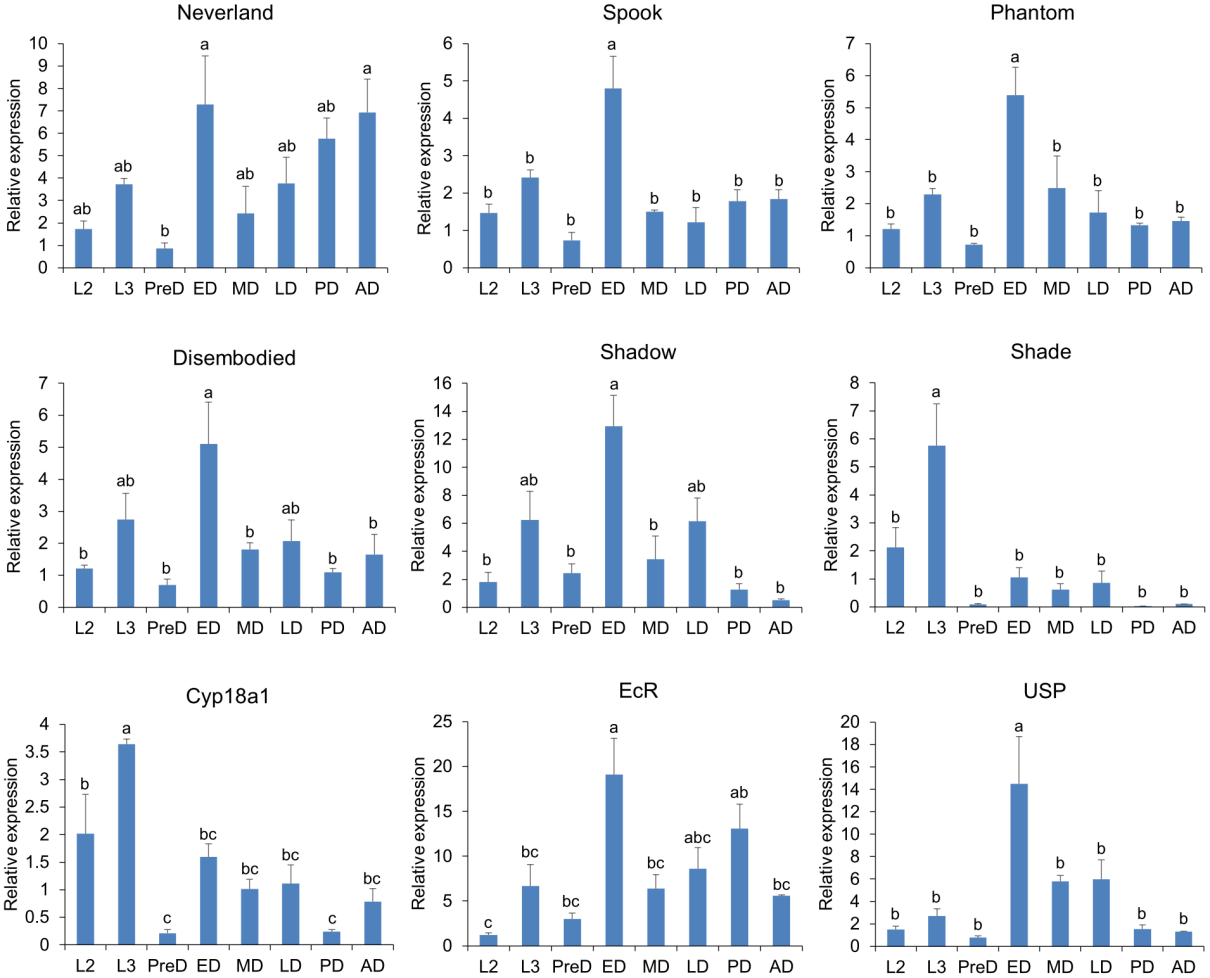
Relative expression of 20E-related genes across the developmental stages of *Bactrocera minax*. L2, second-instar larvae; L3, third-instar larvae; PreD, pre-diapause; ED, early diapause; MD, middle diapause; LD, late diapause; PD, post-diapause; AD, adult. Bars represent means ± SEM. Different letters above the bars indicate significant differences (P < 0.05, Tukey’s test)

The expression patterns of identified 20E-related genes across the developmental stages were investigated to understand the potential roles of these genes in *B*. *minax* development (Fig 9). All genes expressed higher in the third-instar larvae than did in the second-instar larvae, which is expected to meet the requirement of metamorphosis. After pupation, the expressions of almost all genes decreased to lowest levels prior to diapause occurrence. Accordingly, the 20E titer declined to the lowest level at this stage, implying that the low 20E titer may be the prerequisite for diapause occurrence as the injection of exogenous 20E at this stage averted diapause and advanced adult emergence [28]. Once diapause initiated, the expressions of all genes, except *Shade* and *Cyp18a1*, significantly increased to highest levels, and the 20E titer elevated as well [28]. In addition, injection of exogenous 20E at this stage did not advance adult emergence (data not shown), indicating that once initiated, the diapause is barely affected by 20E titer. Then, the expressions of these genes, except *Shade* and *Cyp18a1*, decreased again at the middle-diapause stage and did not show significant variation thereafter. Interestingly, the expression of *Shade*, which is responsible for the last step in 20E biosynthesis, remained relatively lower across the diapause stages, suggesting that *Shade* may control the rate-limiting step in the 20E biosynthesis pathway. Similarly, the expression of *Cyp18a1* also remained relatively lower across the diapause stages, probably contributing to the accumulation of 20E, which reaches the highest level at late pupal stage [28].

**Fig 10:**
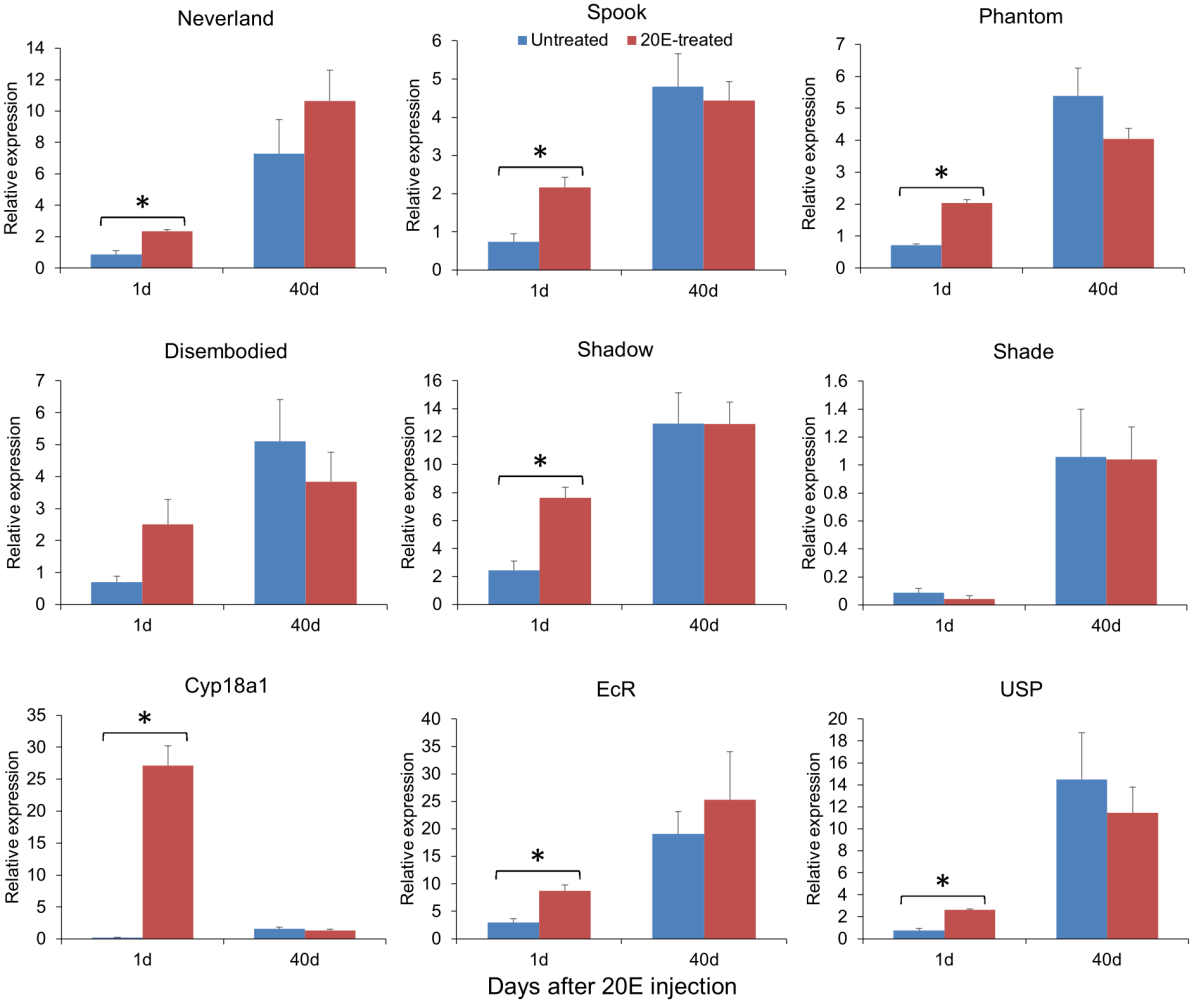
Relative expression of 20E-related genes between 20E-treated and untreated *Bactrocera minax* pupae. Bars represent means ± SEM. Asterisks indicate significant differences in relative expression (P < 0.05, unpaired t-test).

The expressions of these genes were compared between 20E-treated and untreated pupae to understand the effects of exogenous 20E application on 20E biosynthesis and signaling (Fig 10). One day after injection, the expressions of EcR and USP were significant higher in 20E-treated pupae compared to those in untreated ones, implying that ecdysone receptors are involved in 20E signaling and thus averting the diapause. Likewise, the expressions of most genes in 20E biosynthesis pathway were up-regulated in 20E-treated pupae. The *shade*, however, was down-regulated, albeit not significant. As *Shade* is presumed to control rate-limiting step in 20E biosynthesis pathway, exogenous 20E application may suppress endogenous 20E biosynthesis. Interestingly, the expression of Cyp18a1 was sharply up-regulated in 20E-treated pupae, probably in order to degrade the surplus exogenous 20E. Forty days after injection, the effect of 20E disappeared as all genes did not present significantly different expression.

## Supporting Information

S1 Fig Saturation curve of transcriptome sequencing reads for *Bactrocera minax*. (TIF)

S2 Fig Length and number distribution of *Bactrocera minax* transcriptome unigenes. (TIF)

S3 Fig Distribution of *Bactrocera minax* unigene sequences among Kyoto Encyclopedia of Genes and Genomes (KEGG) pathways. The top 40 most highly represented pathways are shown.
(TIF)

S4 Fig Amino acid sequence alignment for ferritin subunit heavy chain homologs (HCH)(A) and light chain homologs (LCH)(B) from *Bactrocera minax* (BM) and other insects. AT, *Asobara tabida*. BD, *Bactrocera dorsalis*. CC, *Ceratitis capitata*. DM, *Drosophila melanogaster*. C residues involved in inter- and intra-subunit disulfide bonds are shaded in yellow. Residues at the ferroxidase center are shaded in red. Residues engaged in the salt bridges and pi-cation interactions are shaded in green. Putative N-glycosylation sites (N-X-S/T) are shaded in grey. Putative signal peptide in HCH and LCH subunits of *B*. *minax* were underlined.
(TIF)

S1 Table Primer sequences used for qRT-PCR analysis of selected genes. (DOCX)

S2 Table Statistics of GO categories from *Bactrocera minax* transcriptomic sequences. (DOCX)

S3 Table Number of unigenes assigned with insulin-related Gene Ontology (GO) terms. (DOCX)

S4 Table Distribution of simple sequence repeat (SSR) types found in the *Bactrocera minax* transcriptome unigenes. (DOCX)

S5 Table Unigene sequences for Hsps identified in *Bactrocera minax* transcriptome. (XLSX)

S6 Table Unigene sequences for GSTs identified in *Bactrocera minax* transcriptome. (XLSX)

S7 Table Unigene sequences for ferritin subunits identified in *Bactrocera minax* transcriptome. (XLSX)

S8 Table Unigene sequences for enzymes in 20E biosynthesis pathway and ecdysone receptors identified in *Bactrocera minax* transcriptome. (XLSX)

## References

[1] Metzker ML. Sequencing technologies—the next generation. Nat Rev Genet. 2010; 11: 31–46. 10.1038/nrg2626 19997069

[2] Kozarewa I, Ning Z, Quail MA, Sanders MJ, Berriman M, Turner DJ. Amplification-free Illumina sequencing-library preparation facilitates improved mapping and assembly of (G+C)-biased genomes. Nat Methods. 2009; 6: 291–295. 10.1038/nmeth.1311 19287394 PMC2664327

[3] Yassour M, Kaplan T, Fraser HB, Levin JZ, Pfiffner J, Adiconis X, et al Ab initio construction of a eukaryotic transcriptome by massively parallel mRNA sequencing. P Natl Acad Sci USA. 2009; 106: 3264–3269. 10.1073/pnas.0812841106 PMC2638735 19208812

[4] Wang XW, Luan JB, Li JM, Bao YY, Zhang CX, Liu SS. *De novo* characterization of a whitefly transcriptome and analysis of its gene expression during development. BMC Genomics. 2010; 11: 400 10.1186/1471-2164-11-400 20573269 PMC2898760

[5] Wei DD, Chen EH, Ding TB, Chen SC, Dou W, Wang JJ. *De novo* assembly, gene annotation, and marker discovery in stored-product pest *Liposcelis entomophila* (Enderlein) using transcriptome sequences. PLoS ONE. 2013; 8: e80046 10.1371/journal.pone.0080046 24244605 PMC3828239

[6] Shen GM, Dou W, Niu JZ, Jiang HB, Yang WJ, Jia FX, et al Transcriptome analysis of the oriental fruit fly (*Bactrocera dorsalis*). PLoS ONE. 2011; 6: e29127 10.1371/journal.pone.0029127 22195006 PMC3240649

[7] Lin T, Cai Z, Wu H. Transcriptome analysis of the Japanese pine sawyer beetle, *Monochamus alternatus* (Coleoptera: Cerambycidae) by high-throughput Illumina sequencing. J Asia Pac Entomol. 2015; 18: 439–445.

[8] Zhou X, Qian K, Tong Y, Zhu JJ, Qiu X, Zeng X. *De novo* transcriptome of the hemimetabolous German cockroach (*Blattella germanica*). PLoS ONE. 2014; 9: e106932 10.1371/journal.pone.0106932 25265537 PMC4180286

[9] Zhang M, Yu H, Yang Y, Song C, Hu X, Zhang G. Analysis of the transcriptome of blowfly *Chrysomya megacephala* (Fabricius) larvae in responses to different edible oils. PLoS ONE. 2013; 8: e63168 10.1371/journal.pone.0063168 23690992 PMC3653882

[10] Zane L, Bargelloni L, Patarnello T. Strategies for microsatellite isolation: A review. Mol Ecol. 2002; 11: 1–16. 11903900 10.1046/j.0962-1083.2001.01418.x

[11] Selkoe KA, Toonen RJ. Microsatellites for ecologists: A practical guide to using and evaluating microsatellite markers. Ecol Lett. 2006; 9: 615–629. 16643306 10.1111/j.1461-0248.2006.00889.x

[12] Dorji C, Clarke AR, Drew RAI, Fletcher BS, Loday P, Mahat K, et al Seasonal phenology of *Bactrocera minax* (Diptera: Tephritidae) in western Bhutan. B Entomol Res. 2006; 96: 531–538. 17092364

[13] Drew RAI, Dorji C, Romig MC, Loday P. Attractiveness of various combinations of colors and shapes to females and males of *Bactrocera minax* (Diptera: Tephritidae) in a commercial mandarin grove in Bhutan. J Econ Entomol. 2006; 99: 1651–1656. 17066795 10.1603/0022-0493-99.5.1651

[14] Wang XJ, Luo LY. Research progress in the Chinese citrus fruit fly. Entomol Knowl. 1995; 32: 310–315.

[15] Allwood AJ, Chinajariyawong A, Kritsaneepaiboon S, Drew RAI, Hamacek EL, Hancock DL, et al Host plant records for fruit flies (Diptera: Tephritidae) in Southeast Asia. Raffles B Zool. 1999; supplement No. 7: 1–92.

[16] Dhillon MK, Singh R, Naresh JS, Sharma HC. The melon fruit fly, *Bactrocera cucurbitae*: A review of its biology and management. J Insect Sci. 2005; 5: 40 17119622 10.1093/jis/5.1.40 PMC1615247

[17] Han P, Wang X, Niu CY, Dong YC, Zhu JQ, Desneux N. Population dynamics, phenology, and overwintering of *Bactrocera dorsalis* (Diptera: Tephritidae) in Hubei Province, China. J Pest Sci. 2011; 84: 289–295.

[18] Nardi F, Carapelli A, Dallai R, Roderick GK, Frati F. Population structure and colonization history of the olive fly, *Bactrocera oleae* (Diptera, Tephritidae). Mol Ecol. 2005; 14: 2729–2738. 16029474 10.1111/j.1365-294X.2005.02610.x

[19] van Schoubroeck F (1999) Learning to fight a fly: Developing citrus IPM in Bhutan [Ph.D.dissertation]. Wageningen, the Netherlands: Wageningen University and Research Centre. 200 p.

[20] Chen EH, Dou W, Hu F, Tang S, Zhao ZM, Wang JJ. Purification and biochemical characterization of glutathione S-transferases in *Bactrocera minax* (Diptera: Tephritidae). Fla Entomol. 2012; 95: 593–601.

[21] Dong YC, Wang ZJ, Clarke AR, Pereira R, Desneux N, Niu CY. Pupal diapause development and termination is driven by low temperature chilling in *Bactrocera minax*. J Pest Sci. 2013; 86: 429–436.

[22] Zhang Y, Zhang ZM. Occurrence and integrated control methods of Chinese citrus fly *Bactrocera minax*. Bull Agric Sci Technol. 2005; 2: 22–23.

[23] Zhang YA. Citrus fruit flies of Sichuan Province (China). EPPO Bull. 1989; 19: 649–654.

[24] Wang XL, Zhang RJ. Review on biology, ecology and control of *Bactrocera (Tetradacus) minax* Enderlein. J Environ Entomol. 2009; 31: 73–79.

[25] Liu HQ, Jiang GF, Zhang YF, Chen F, Li XJ, Yue JS, et al Effect of six insecticides on three populations of *Bactrocera* (tetradacus) *minax* (Diptera: Tephritidae). Curr Pharm Biotechno. 2015; 16: 77–83. 10.2174/138920101601150105105751 25564253

[26] Dong YC, Wan L, Pereira R, Desneux N, Niu CY. Feeding and mating behaviour of Chinese citrus fly *Bactrocera minax* (Diptera, Tephritidae) in the field. J Pest Sci. 2014; 87: 647–657.

[27] Zhang B, Nardi F, Hull-Sanders H, Wan XW, Liu YH. The complete nucleotide sequence of the mitochondrial genome of *Bactrocera minax* (Diptera: Tephritidae). PLoS ONE. 2014; 9: e100558 10.1371/journal.pone.0100558 24964138 PMC4070923

[28] Wang J, Zhou HY, Zhao ZM, Liu YH. Effects of juvenile hormone analogue and ecdysteroid on adult eclosion of the fruit fly *Bactrocera minax* (Diptera: Tephritidae). J Econ Entomol. 2014; 107: 1519–1525. 25195444 10.1603/ec13539

[29] Wang AL, Yao ZC, Zheng WW, Zhang HY. Bacterial communities in the gut and reproductive organs of *Bactrocera minax* (diptera: Tephritidae) based on 454 pyrosequencing. PLoS ONE. 2014; 9: e106988 10.1371/journal.pone.0106988 25215866 PMC4162550

[30] Lu ZC, Wang LH, Zhang GF, Wan FH, Guo JY, Yu H, et al Three heat shock protein genes from *Bactrocera* (tetradacus) *minax* Enderlein: Gene cloning, characterization, and association with diapause. Neotrop Entomol. 2014; 43: 362–372. 10.1007/s13744-014-0216-y 27193815

[31] Lu ZC, Wang LH, Dai RL, Zhang GF, Guo JY, Wan FH. Evaluation of endogenous reference genes of *Bactrocera* (tetradacus) *minax* by gene expression profiling under various experimental conditions. Fla Entomol. 2014; 97: 597–604.

[32] Dong YC, Desneux N, Lei CL, Niu CY. Transcriptome characterization analysis of *Bactrocera minax* and new insights into its pupal diapause development with gene expression analysis. Int J Biol Sci. 2014; 10: 1051–1063. 10.7150/ijbs.9438 25285037 PMC4183925

[33] Gilbert LI. Halloween genes encode P450 enzymes that mediate steroid hormone biosynthesis in *Drosophila melanogaster*. Mol Cell Endocrinol. 2004; 215: 1–10. 15026169 10.1016/j.mce.2003.11.003

[34] Guittard E, Blais C, Maria A, Parvy JP, Pasricha S, Lumb C, et al CYP18A1, a key enzyme of Drosophila steroid hormone inactivation, is essential for metamorphosis. Dev Biol. 2011; 349: 35–45. 10.1016/j.ydbio.2010.09.023 20932968

[35] Spindler KD, Honl C, Tremmel C, Braun S, Ruff H, Spindler-Barth M. Ecdysteroid hormone action. Cell Mol Life Sci. 2009; 66: 3837–3850. 10.1007/s00018-009-0112-5 19669094 PMC11115491

[36] Koelle MR, Talbot WS, Segraves WA, Bender MT, Cherbas P, Hogness DS. The Drosophila EcR gene encodes an ecdysone receptor, a new member of the steroid-receptor superfamily. Cell. 1991; 67: 59–77. 1913820 10.1016/0092-8674(91)90572-g

[37] Yao TP, Segraves WA, Oro AE, Mckeown M, Evans RM. Drosophila ultraspiracle modulates ecdysone receptor function via heterodimer formation. Cell. 1992; 71: 63–72. 1327536 10.1016/0092-8674(92)90266-f

[38] Swevers L, Cherbas L, Cherbas P, Iatrou K. Bombyx EcR (BmEcR) and Bombyx USP (BmCF1) combine to form a functional ecdysone receptor. Insect Biochem Molec. 1996; 26: 217–221. 10.1016/0965-1748(95)00097-6 8900593

[39] Feder ME, Hofmann GE. Heat-shock proteins, molecular chaperones, and the stress response: Evolutionary and ecological physiology. Annu Rev Physiol. 1999; 61: 243–282. 10099689 10.1146/annurev.physiol.61.1.243

[40] Enayati AA, Ranson H, Hemingway J. Insect glutathione transferases and insecticide resistance. Insect Mol Biol. 2005; 14: 3–8. 15663770 10.1111/j.1365-2583.2004.00529.x

[41] Nichol H, Law JH, Winzerling JJ. Iron metabolism in insects. Annu Rev Entomol. 2002; 47: 535–559. 11729084 10.1146/annurev.ento.47.091201.145237

[42] King AM, MacRae TH. Insect heat shock proteins during stress and diapause. Annu Rev Entomol. 2015; 60: 59–75. 10.1146/annurev-ento-011613-162107 25341107

[43] Zhang Q, Lu YX, Xu WH. Proteomic and metabolomic profiles of larval hemolymph associated with diapause in the cotton bollworm, *Helicoverpa armigera*. BMC Genomics. 2013; 14: 751 10.1186/1471-2164-14-751 24180224 PMC4046812

[44] Bao B, Xu WH. Identification of gene expression changes associated with the initiation of diapause in the brain of the cotton bollworm, *Helicoverpa armigera*. BMC Genomics. 2011; 12: 224 10.1186/1471-2164-12-224 21569297 PMC3277317

[45] Patel RK, Jain M. NGS QC Toolkit: a toolkit for quality control of next generation sequencing data. PLoS ONE. 2012; 7: e30619 10.1371/journal.pone.0030619 22312429 PMC3270013

[46] Kopylova E, Noe L, Touzet H. SortMeRNA: fast and accurate filtering of ribosomal RNAs in metatranscriptomic data. Bioinformatics. 2012; 28: 3211–3217. 10.1093/bioinformatics/bts611 23071270

[47] Grabherr MG, Haas BJ, Yassour M, Levin JZ, Thompson DA, Amit I, et al Full-length transcriptome assembly from RNA-Seq data without a reference genome. Nat Biotechnol. 2011; 29: 644–652. 10.1038/nbt.1883 21572440 PMC3571712

[48] Gotz S, Garcia-Gomez JM, Terol J, Williams TD, Nagaraj SH, Nueda MJ, et al High-throughput functional annotation and data mining with the Blast2GO suite. Nucleic Acids Res. 2008; 36: 3420–3435. 10.1093/nar/gkn176 18445632 PMC2425479

[49] Tatusov RL, Natale DA, Garkavtsev IV, Tatusova TA, Shankavaram UT, Rao BS, et al The COG database: new developments in phylogenetic classification of proteins from complete genomes. Nucleic Acids Res. 2001; 29: 22–28. 11125040 10.1093/nar/29.1.22 PMC29819

[50] Sharma PC, Grover A, Kahl G. Mining microsatellites in eukaryotic genomes. Trends Biotechnol. 2007; 25: 490–498. 17945369 10.1016/j.tibtech.2007.07.013

[51] Tamura K, Dudley J, Nei M, Kumar S. MEGA4: Molecular Evolutionary Genetics Analysis (MEGA) software version 4.0. Mol Biol Evol. 2007; 24: 1596–1599. 17488738 10.1093/molbev/msm092

[52] Brown JB, Boley N, Eisman R, May GE, Stoiber MH, Duff MO, et al Diversity and dynamics of the *Drosophila* transcriptome. Nature. 2014; 512: 393–399. 24670639 10.1038/nature12962 PMC4152413

[53] Ashburner M, Ball CA, Blake JA, Botstein D, Butler H, Cherry JM, et al Gene ontology: tool for the unification of biology. The Gene Ontology Consortium. Nat Genet. 2000; 25: 25–29. 10802651 10.1038/75556 PMC3037419

[54] Sim C, Denlinger DL. Insulin signaling and the regulation of insect diapause. Front Physiol. 2013; 4: 189 10.3389/fphys.2013.00189 23885240 PMC3717507

[55] Kanehisa M, Goto S. KEGG: kyoto encyclopedia of genes and genomes. Nucleic Acids Res. 2000; 28: 27–30. 10592173 10.1093/nar/28.1.27 PMC102409

[56] Denlinger D, Yocum G, Rinehart J. Hormonal Control of Diapause In: Gilbert LI, editor. Insect Endocrinology. Waltham, MA: Elsevier 2011 pp. 430–463.

[57] Young SC, Yeh WL, Gu SH. Transcriptional regulation of the PTTH receptor in prothoracic glands of the silkworm, *Bombyx mori*. J Insect Physiol. 2012; 58: 102–109. 10.1016/j.jinsphys.2011.10.005 22085674

[58] Gao LZ, Liu YH, Wan XW, Wang J, Hong F. Screening of microsatellite markers in *Bactrocera minax* (Diptera: Tephritidae). Sci Agric Sin. 2013; 46: 3285–3292.

[59] Sørensen JG, Kristensen TN, Loeschcke V. The evolutionary and ecological role of heat shock proteins. Ecol Lett. 2003; 6: 1025–1037.

[60] Rinehart JP, Li A, Yocum GD, Robich RM, Hayward SAL, Denlinger DL. Up-regulation of heat shock proteins is essentail for cold survival during insect diapause. P Natl Acad Sci USA. 2007; 104: 11130–11137. 10.1073/pnas.0703538104 PMC2040864 17522254

[61] Li J, Buchner J. Structure, function and regulation of the hsp90 machinery. Biomed J. 2013; 36: 106–117. 10.4103/2319-4170.113230 23806880

[62] Mayer MP, Bukau B. Hsp70 chaperones: Cellular functions and molecular mechanism. Cell Mol Life Sci. 2005; 62: 670–684. 15770419 10.1007/s00018-004-4464-6 PMC2773841

[63] Jones M, Gupta RS, Englesberg E. Enhancement in amount of P1 (hsp60) in mutants of Chinese hamster ovary (CHO-K1) cells exhibiting increases in the A system of amino acid transport. P Natl Acad Sci USA. 1994; 91: 858–862. 10.1073/pnas.91.3.858 PMC521411 7905632

[64] Kaufman BA, Kolesar JE, Perlman PS, Butow RA. A function for the mitochondrial chaperonin Hsp60 in the structure and transmission of mitochondrial DNA nucleoids in *Saccharomyces cerevisiae*. J Cell Biol. 2003; 163: 457–461. 14597775 10.1083/jcb.200306132 PMC2173642

[65] Ikawa S, Weinberg RA. An interaction between p21ras and heat shock protein hsp60, a chaperonin. P Natl Acad Sci USA. 1992; 89: 2012–2016. 10.1073/pnas.89.6.2012 PMC48586 1347942

[66] Koll H, Guiard B, Rassow J, Ostermann J, Horwich AL, Neupert W, et al Antifolding activity of hsp60 couples protein import into the mitochondrial matrix with export to the intermembrane space. Cell. 1992; 68: 1163–1175. 1347713 10.1016/0092-8674(92)90086-r

[67] Qiu XB, Shao YM, Miao S, Wang L. The diversity of the DnaJ/Hsp40 family, the crucial partners for Hsp70 chaperones. Cell Mol Life Sci. 2006; 63: 2560–2570. 16952052 10.1007/s00018-006-6192-6 PMC11136209

[68] Samali A, Cai J, Zhivotovsky B, Jones DP, Orrenius S. Presence of a pre-apoptotic complex of pro-caspase-3, Hsp60 and Hsp10 in the mitochondrial fraction of jurkat cells. EMBO J. 1999; 18: 2040–2048. 10205158 10.1093/emboj/18.8.2040 PMC1171288

[69] Jakob U, Gaestel M, Engel K, Buchner J. Small heat shock proteins are molecular chaperones. J Biol Chem. 1993; 268: 1517–1520. 8093612

[70] Hayes JD, Flanagan JU, Jowsey IR. Glutathione transferases. Annu Rev Pharmacol Toxico. 2005; 45: 51–88. 10.1146/annurev.pharmtox.45.120403.095857 15822171

[71] Tu CP, Akgul B. Drosophila glutathione S-transferases. Methods Enzymol. 2005; 401: 204–226. 16399388 10.1016/S0076-6879(05)01013-X

[72] Ranson H, Claudianos C, Ortelli F, Abgrall C, Hemingway J, Sharakhova MV, et al Evolution of supergene families associated with insecticide resistance. Science. 2002; 298: 179–181. 12364796 10.1126/science.1076781

[73] Lumjuan N, Rajatileka S, Changsom D, Wicheer J, Leelapat P, Prapanthadara LA, et al The role of the *Aedes aegypti* Epsilon glutathione transferases in conferring resistance to DDT and pyrethroid insecticides. Insect Biochem Molec. 2011; 41: 203–209. 10.1016/j.ibmb.2010.12.005 21195177

[74] Tu X, Wang J, Hao K, Whitman DW, Fan Y, Cao G, et al Transcriptomic and proteomic analysis of pre-diapause and non-diapause eggs of migratory locust, *Locusta migratoria* L. (Orthoptera: Acridoidea). Sci Rep. 2015; 5: 11402 10.1038/srep11402 26091374 PMC4650673

[75] Ragland GJ, Denlinger DL, Hahn DA. Mechanisms of suspended animation are revealed by transcript profiling of diapause in the flesh fly. P Natl Acad Sci USA. 2010; 107: 14909–14914. 10.1073/pnas.1007075107 PMC2930464 20668242

[76] Andrews NC. Forging a field: the golden age of iron biology. Blood. 2008; 112: 219–230. 10.1182/blood-2007-12-077388 18606887 PMC2442739

[77] Hower V, Mendes P, Torti FM, Laubenbacher R, Akman S, Shulaev V, et al A general map of iron metabolism and tissue-specific subnetworks. Mol Biosyst. 2009; 5: 422–443. 10.1039/b816714c 19381358 PMC2680238

[78] Hamburger AE, West AP Jr, Hamburger ZA, Hamburger P, Bjorkman PJ. Crystal structure of a secreted insect ferritin reveals a symmetrical arrangement of heavy and light chains. J Mol Biol. 2005; 349: 558–569. 15896348 10.1016/j.jmb.2005.03.074

[79] Jiang XZ, Cong L, Niu JZ, Dou W, Wang JJ. Alternative splicing contributes to the coordinated regulation of ferritin subunit levels in *Bactrocera dorsalis* (Hendel). Sci Rep. 2014; 4: 4806 10.1038/srep04806 24763285 PMC3999511

[80] Yoshiyama-Yanagawa T, Enya S, Shimada-Niwa Y, Yaguchi S, Haramoto Y, Matsuya T, et al The conserved Rieske oxygenase DAF-36/Neverland is a novel cholesterol-metabolizing enzyme. J Biol Chem. 2011; 286: 25756–25762. 10.1074/jbc.M111.244384 21632547 PMC3138242

[81] Tran HT, Shaaban S, Askari HB, Walfish PG, Raikhel AS, Butt TR. Requirement of co-factors for the ligand-mediated activity, of the insect ecdysteroid receptor in yeast. J Mol Endocrinol. 2001; 27: 191–209. 11564603 10.1677/jme.0.0270191

