# Supplementary figures and images for: De novo Transcriptome Analysis of Chinese Citrus Fly, Bactrocera minax (Diptera: Tephritidae), by High-Throughput Illumina Sequencing
Source: PLoS One. 2016 Jun 22;11(6):e0157656. doi: 10.1371/journal.pone.0157656 (PMC4917245; doi:10.1371/journal.pone.0157656)

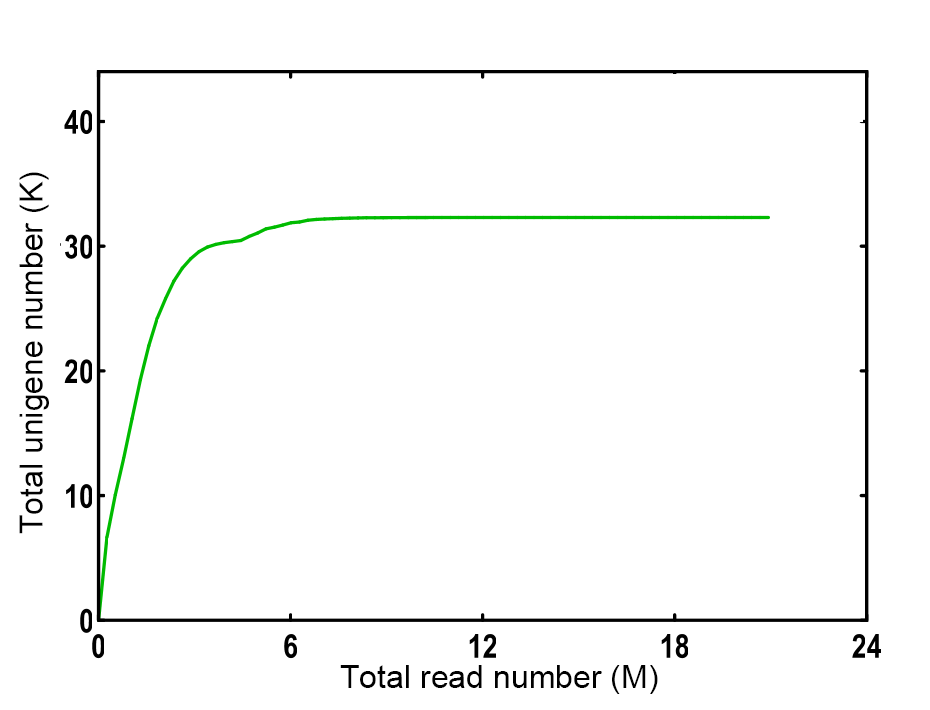

Supplement: S1 Fig — (TIF) [file pone.0157656.s001.tif]

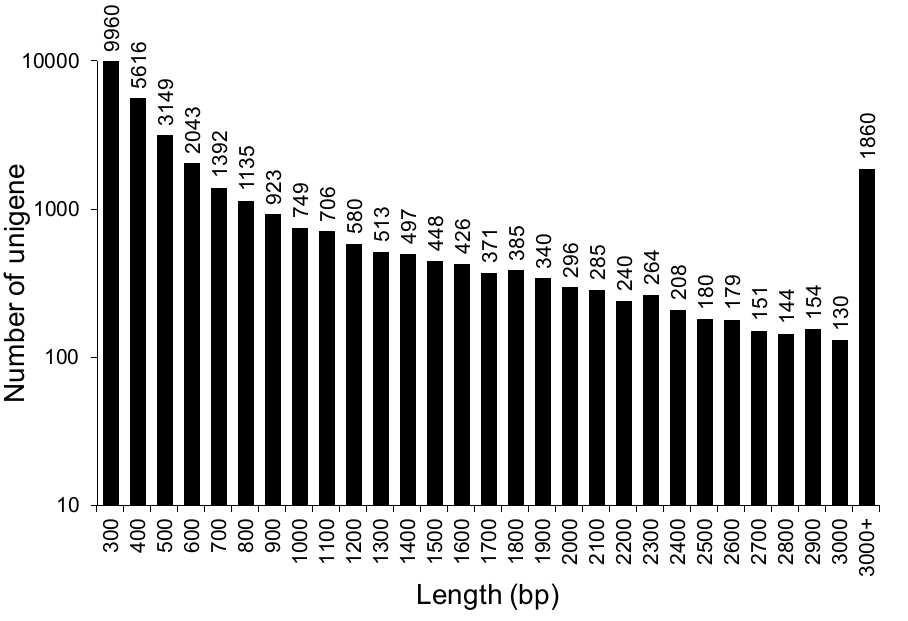

Supplement: S2 Fig — (TIF) [file pone.0157656.s002.tif]

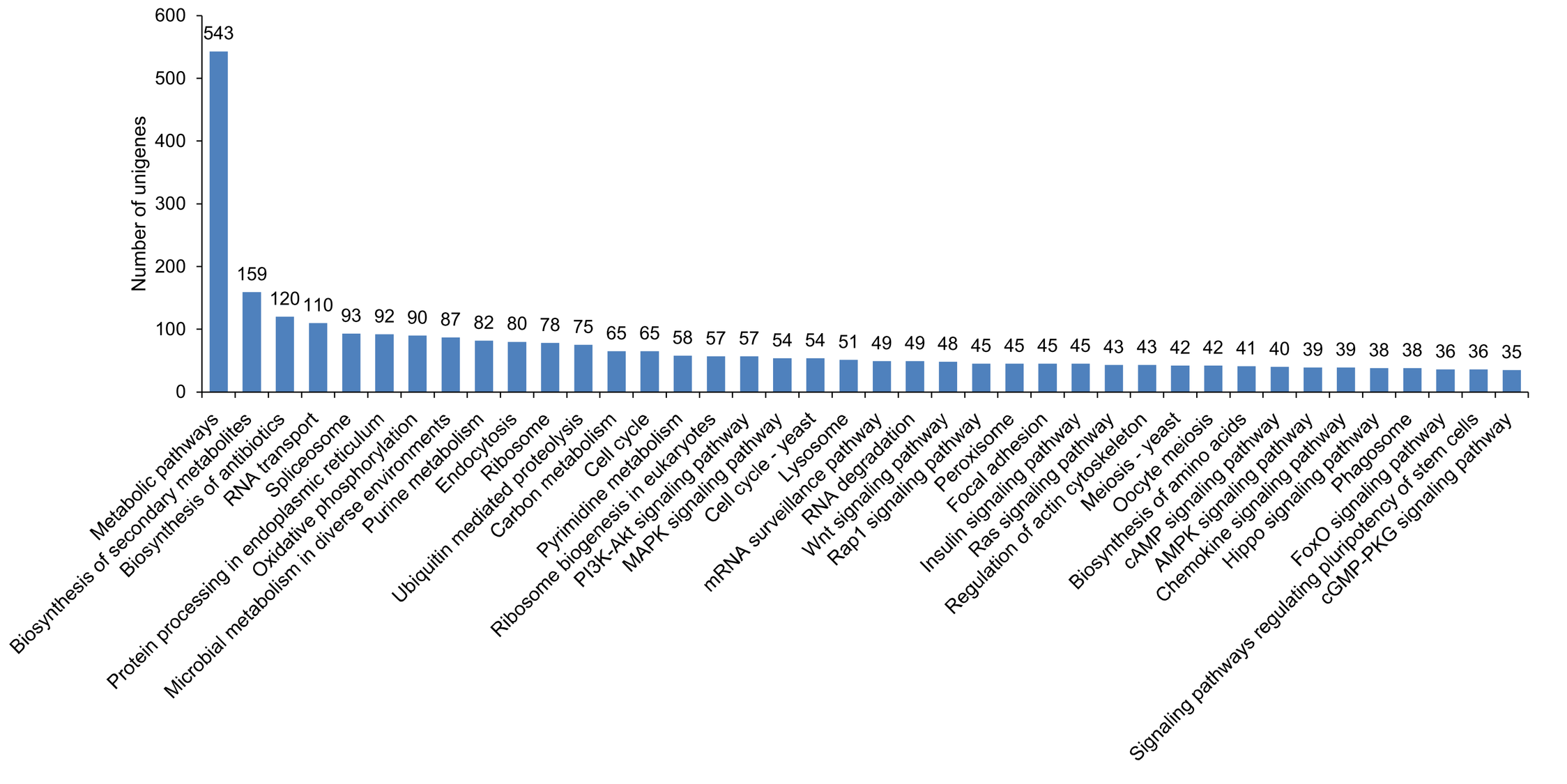

Supplement: S3 Fig — The top 40 most highly represented pathways are shown. (TIF) [file pone.0157656.s003.tif]

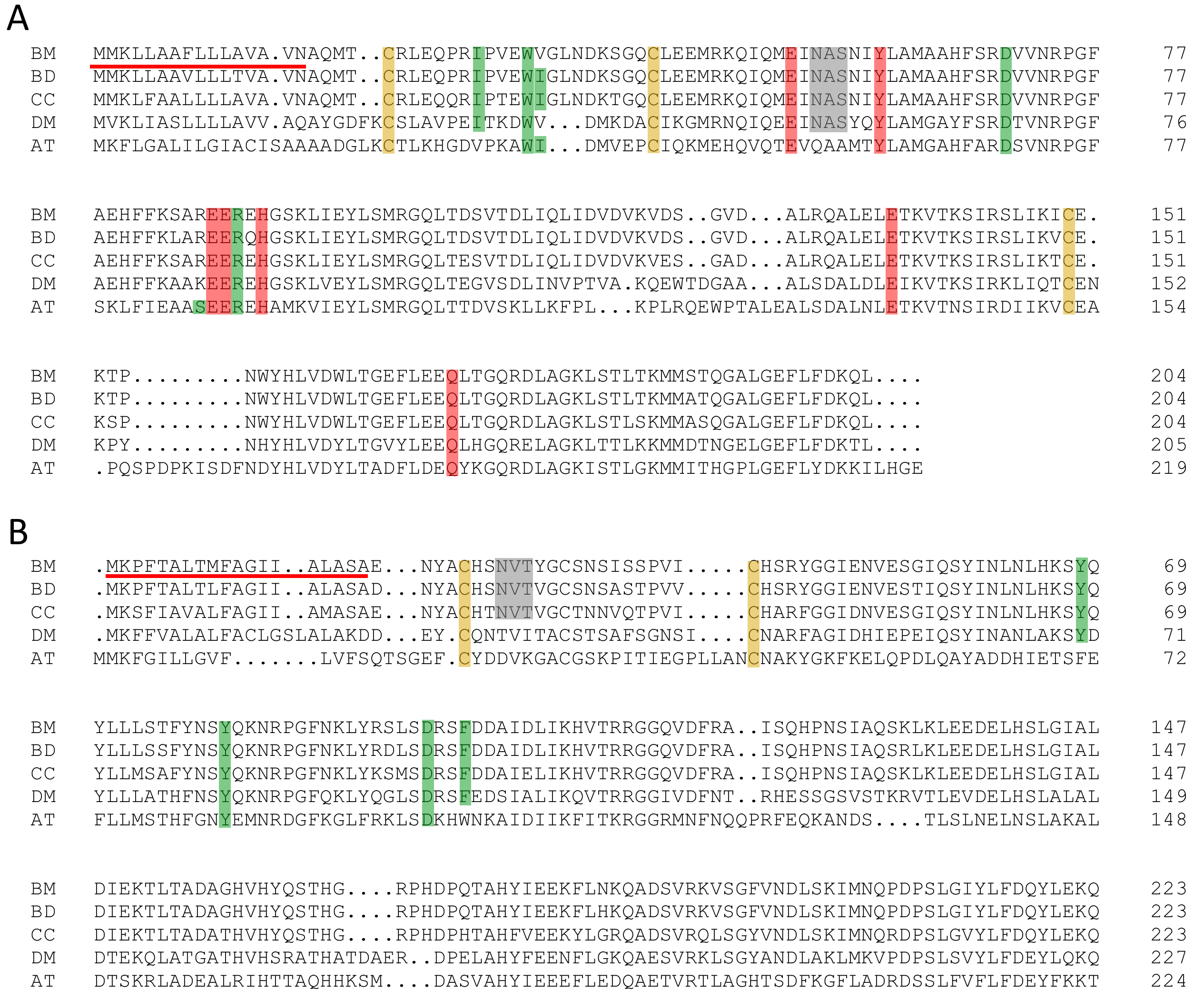

Supplement: S4 Fig — AT, Asobara tabida. BD, Bactrocera dorsalis. CC, Ceratitis capitata. DM, Drosophila melanogaster. C residues involved in inter- and intra-subunit disulfide bonds are shaded in yellow. Residues at the ferroxidase center are shaded in red. Residues engaged in the salt bridges and pi-cation interactions are shaded in green. Putative N-glycosylation sites (N-X-S/T) are shaded in grey. Putative signal peptide in HCH and LCH subunits of B. minax were underlined. (TIF) [file pone.0157656.s004.tif]
