# Supplementary material for: De novo Transcriptome Analysis of Chinese Citrus Fly, Bactrocera minax (Diptera: Tephritidae), by High-Throughput Illumina Sequencing
Source: PLoS One. 2016 Jun 22;11(6):e0157656. doi: 10.1371/journal.pone.0157656 (PMC4917245; doi:10.1371/journal.pone.0157656)
Supplement: S1 Table — (DOCX) [file pone.0157656.s005.docx]

S1 Table. Primer sequences used for qRT-PCR analysis of selected genes

| Unigene ID | Gene name | Forward primer (5’-3’) | Reverse primer (5’-3’) |
| --- | --- | --- | --- |
| BmUnigene26430 c2 | EcR | CACCATTAAGCCCATCACCG | CACCAGACACAGTTCCTCCT |
| BmUnigene19978 c0 | USP | TGGTGATAGGGCAAGTGGAA | TGCCACGTTGTCTCTCTTCT |
| BmUnigene37082.c0 | Neverland | CCCACTTTTCAGCAGTCCAC | CCAAGTGTACGAATGAAGGTCC |
| BmUnigene33796.c0 | Spook | CCGATAAAGGCGTTTGTTCAAC | ACCAAGGCAGGAAATCAACG |
| BmUnigene22471.c1 | Phantom | ACTTGGTGTTTGGCGTGAAA | CGAGTAGGAAGCGTAGGGAG |
| BmUnigene9752.c0 | Disembodied | TTCTCGTCTATGTCGGACCG | CGATCGCTCCATATTGCTCG |
| BmUnigene26483.c0 | Shadow | ACAAGCAGCACAGTTGTCAG | TGCGCTTTCCACAGATTCAC |
| BmUnigene25907.c0 | Shade | CCATCTGGCAGAAACTACGC | GACCCAACATGAGCGTACAC |
| BmUnigene24695.c0 | Cyp18a1 | AATCGCCAAGAATCGTCACG | GAACAGTTCCTCATCGCGTC |
