# Supplementary material for: De novo Transcriptome Analysis of Chinese Citrus Fly, Bactrocera minax (Diptera: Tephritidae), by High-Throughput Illumina Sequencing
Source: PLoS One. 2016 Jun 22;11(6):e0157656. doi: 10.1371/journal.pone.0157656 (PMC4917245; doi:10.1371/journal.pone.0157656)
Supplement: S2 Table — (DOCX) [file pone.0157656.s006.docx]

S2 Table. Statistics of GO categories for *Bactrocera minax* transcriptome unigenes.

| Ontology | Class | No. of GO term | Percentage (%) |
| --- | --- | --- | --- |
| **Cellular component** |  | **25,452** | **33.36** |
|  | cell part | 5,116 | 6.71 |
|  | cell | 5,089 | 6.67 |
|  | organelle | 3,817 | 5.00 |
|  | macromolecular complex | 2,456 | 3.22 |
|  | membrane | 2,447 | 3.21 |
|  | organelle part | 2,406 | 3.15 |
|  | membrane part | 1,779 | 2.33 |
|  | membrane-enclosed lumen | 560 | 0.73 |
|  | extracellular region | 517 | 0.68 |
|  | synapse | 363 | 0.48 |
|  | extracellular region part | 264 | 0.35 |
|  | synapse part | 256 | 0.34 |
|  | cell junction | 255 | 0.33 |
|  | extracellular matrix | 92 | 0.12 |
|  | extracellular matrix part | 28 | 0.04 |
|  | nucleoid | 4 | 0.01 |
|  | virion | 1 | 0.00 |
|  | virion part | 1 | 0.00 |
| **Molecular function** |  | **12,681** | **16.62** |
|  | binding | 5,030 | 6.59 |
|  | catalytic activity | 4,295 | 5.63 |
|  | transporter activity | 877 | 1.15 |
|  | structural molecule activity | 476 | 0.62 |
|  | nucleic acid binding transcription factor activity | 432 | 0.57 |
|  | molecular transducer activity | 410 | 0.54 |
|  | receptor activity | 405 | 0.53 |
|  | enzyme regulator activity | 384 | 0.50 |
|  | protein binding transcription factor activity | 132 | 0.17 |
|  | electron carrier activity | 127 | 0.17 |
|  | antioxidant activity | 45 | 0.06 |
|  | translation regulator activity | 21 | 0.03 |
|  | channel regulator activity | 16 | 0.02 |
|  | morphogen activity | 14 | 0.02 |
|  | receptor regulator activity | 10 | 0.01 |
|  | metallochaperone activity | 3 | 0.00 |
|  | nutrient reservoir activity | 3 | 0.00 |
|  | protein tag | 1 | 0.00 |
| **Biological process** |  | **3,8159** | **50.02** |
|  | cellular process | 6,726 | 8.82 |
|  | metabolic process | 5,439 | 7.13 |
|  | biological regulation | 3,822 | 5.01 |
|  | developmental process | 3,417 | 4.48 |
|  | multicellular organismal process | 3,029 | 3.97 |
|  | response to stimulus | 2,624 | 3.44 |
|  | cellular component organization or biogenesis | 2,403 | 3.15 |
|  | localization | 2,242 | 2.94 |
|  | establishment of localization | 1,795 | 2.35 |
|  | signaling | 1,471 | 1.93 |
|  | reproduction | 1,271 | 1.67 |
|  | reproductive process | 1,205 | 1.58 |
|  | locomotion | 802 | 1.05 |
|  | multi-organism process | 421 | 0.55 |
|  | immune system process | 303 | 0.40 |
|  | biological adhesion | 298 | 0.39 |
|  | growth | 272 | 0.36 |
|  | cell proliferation | 199 | 0.26 |
|  | death | 193 | 0.25 |
|  | rhythmic process | 131 | 0.17 |
|  | pigmentation | 92 | 0.12 |
|  | cell killing | 2 | 0.00 |
|  | viral reproduction | 2 | 0.00 |
