# Supplementary material for: De novo Transcriptome Analysis of Chinese Citrus Fly, Bactrocera minax (Diptera: Tephritidae), by High-Throughput Illumina Sequencing
Source: PLoS One. 2016 Jun 22;11(6):e0157656. doi: 10.1371/journal.pone.0157656 (PMC4917245; doi:10.1371/journal.pone.0157656)
Supplement: S3 Table — (DOCX) [file pone.0157656.s007.docx]

S3 Table. Number of unigenes assigned with insulin-related Gene Ontology (GO) terms

| Ontology | Class | GO annotation | No. of unigene |
| --- | --- | --- | --- |
| **Cellular component** |  |  | **2** |
|  | insulin receptor complex | GO:0005899 | 2 |
| **Molecular function** |  |  | **10** |
|  | insulin receptor binding | GO:0005158 | 4 |
|  | insulin-activated receptor activity | GO:0005009 | 2 |
|  | insulin-like growth factor binding | GO:0005520 | 3 |
|  | insulin-like growth factor receptor binding | GO:0005159 | 1 |
| **Biological process** |  |  | **33** |
|  | cellular response to insulin stimulus | GO:0032869 | 3 |
|  | insulin receptor signaling pathway | GO:0008286 | 13 |
|  | insulin-like growth factor receptor signaling pathway | GO:0048009 | 4 |
|  | negative regulation of insulin receptor signaling pathway | GO:0046627 | 8 |
|  | negative regulation of insulin secretion | GO:0046676 | 1 |
|  | positive regulation of insulin receptor signaling pathway | GO:0046628 | 1 |
|  | regulation of insulin receptor signaling pathway | GO:0046626 | 2 |
|  | response to insulin | GO:0032868 | 1 |
