# Supplementary material for: De novo Transcriptome Analysis of Chinese Citrus Fly, Bactrocera minax (Diptera: Tephritidae), by High-Throughput Illumina Sequencing
Source: PLoS One. 2016 Jun 22;11(6):e0157656. doi: 10.1371/journal.pone.0157656 (PMC4917245; doi:10.1371/journal.pone.0157656)
Supplement: S4 Table — (DOCX) [file pone.0157656.s008.docx]

S4 Table. Distribution of simple sequence repeat (SSR) types found in the *Bactrocera minax* transcriptome unigenes.

| Repeat motif | Number of repeats | | | | | | | | | | total | Percentage | |
| --- | --- | --- | --- | --- | --- | --- | --- | --- | --- | --- | --- | --- | --- |
|  | 5 | 6 | 7 | 8 | 9 | 10 | 11 | 12 | >12 | |  | (%) | |
| AC/GT |  | 126 | 64 | 49 | 26 | 17 | 20 | 1 | 1 | | 304 | 15.92 | |
| AT/AT |  | 132 | 44 | 37 | 17 | 13 | 3 | 1 |  | | 247 | 12.94 | |
| AG/CT |  | 23 | 8 | 13 | 6 | 6 | 8 | 1 | 1 | | 66 | 3.46 | |
| CG/CG |  | 3 |  |  |  |  |  |  |  | | 3 | 0.16 | |
| AAC/GTT | 269 | 187 | 100 | 1 |  |  |  |  |  | | 557 | 29.18 | |
| AGC/CTG | 118 | 51 | 31 | 1 |  | 1 |  |  |  | | 202 | 10.58 | |
| ACC/GGT | 74 | 30 | 16 |  |  |  |  |  |  | | 120 | 6.29 | |
| ATC/ATG | 44 | 23 | 11 | 4 |  |  |  |  |  | | 82 | 4.30 | |
| AAT/ATT | 35 | 14 | 6 | 3 |  |  |  |  |  | | 58 | 3.04 | |
| CCG/CGG | 35 | 15 | 4 | 1 |  | 1 |  |  |  | | 56 | 2.93 | |
| AAG/CTT | 32 | 10 | 4 | 1 | 1 |  |  |  |  | | 48 | 2.51 | |
| ACG/CGT | 14 | 13 | 7 | 3 |  | 1 |  |  |  | | 38 | 1.99 | |
| ACT/AGT | 17 | 6 | 3 | 3 |  |  |  |  |  | | 29 | 1.52 | |
| AGG/CCT | 11 | 2 | 2 |  |  |  |  |  |  | | 15 | 0.79 | |
| ACAT/ATGT | 38 | 4 |  | 1 |  |  |  |  |  | | 43 | 2.25 | |
| AAAC/GTTT | 2 |  |  | 1 |  |  |  |  |  | | 3 | 0.16 | |
| AAAT/ATTT | 3 |  |  |  |  |  |  |  |  | | 3 | 0.16 | |
| AAAG/CTTT | 2 |  |  |  |  |  |  |  |  | | 2 | 0.10 | |
| AACC/GGTT | 1 |  |  | 1 |  |  |  |  |  | | 2 | 0.10 | |
| ACTG/AGTC | 1 |  |  |  |  | 1 |  |  |  | | 2 | 0.10 | |
| AGCC/CTGG | 1 | 1 |  |  |  |  |  |  |  | | 2 | 0.10 | |
| AAGG/CCTT | 1 |  |  |  |  |  |  |  |  | | 1 | 0.05 | |
| AAGT/ACTT | 1 |  |  |  |  |  |  |  |  | | 1 | 0.05 | |
| AATG/ATTC | 1 |  |  |  |  |  |  |  |  | | 1 | 0.05 | |
| AATT/AATT | 1 |  |  |  |  |  |  |  |  | | 1 | 0.05 | |
| ACAG/CTGT |  | 1 |  |  |  |  |  |  |  | | 1 | 0.05 | |
| ACCG/CGGT |  |  |  |  |  | 1 |  |  |  | | 1 | 0.05 | |
| ACGG/CCGT | 1 |  |  |  |  |  |  |  |  | | 1 | 0.05 | |
| AGAT/ATCT | 1 |  |  |  |  |  |  |  |  | | 1 | 0.05 | |
| AGGC/CCTG |  |  | 1 |  |  |  |  |  |  | | 1 | 0.05 | |
| AGCTC/AGCTG | 2 |  |  |  |  |  |  |  |  | 2 | | 0.10 |  |
| AAAGC/CTTTG |  | 1 |  |  |  |  |  |  |  | 1 | | 0.05 |  |
| AACTG/AGTTC | 1 |  |  |  |  |  |  |  |  | 1 | | 0.05 |  |
| AATAC/ATTGT |  | 1 |  |  |  |  |  |  |  | 1 | | 0.05 |  |
| AGCAT/ATGCT | 1 |  |  |  |  |  |  |  |  | 1 | | 0.05 |  |
| AAACAC/GTGTTT | 1 |  |  |  |  |  |  |  |  | 1 | | 0.05 |  |
| AAACAG/CTGTTT | 1 |  |  |  |  |  |  |  |  | 1 | | 0.05 |  |
| AACACC/GGTGTT | 1 |  |  |  |  |  |  |  |  | 1 | | 0.05 |  |
| AACATC/ATGTTG |  | 1 |  |  |  |  |  |  |  | 1 | | 0.05 |  |
| AACCTG/AGGTTC | 1 |  |  |  |  |  |  |  |  | 1 | | 0.05 |  |
| AACGGG/CCCGTT | 1 |  |  |  |  |  |  |  |  | 1 | | 0.05 |  |
| AAGGCG/CCTTCG | 1 |  |  |  |  |  |  |  |  | 1 | | 0.05 |  |
| AATCAG/ATTCTG |  | 1 |  |  |  |  |  |  |  | 1 | | 0.05 |  |
| AATCAT/ATGATT |  |  | 1 |  |  |  |  |  |  | 1 | | 0.05 |  |
| AATCCC/ATTGGG | 1 |  |  |  |  |  |  |  |  | 1 | | 0.05 |  |
| AATTAC/AATTGT | 1 |  |  |  |  |  |  |  |  | 1 | | 0.05 |  |
| ACAGTC/ACTGTG | 1 |  |  |  |  |  |  |  |  | 1 | | 0.05 |  |
